# TDP-43 dysregulation and neuromuscular junction disruption in amyotrophic lateral sclerosis

**DOI:** 10.1186/s40035-022-00331-z

**Published:** 2022-12-27

**Authors:** Sarah Lépine, Maria José Castellanos-Montiel, Thomas Martin Durcan

**Affiliations:** 1 The Neuro’s Early Drug Discovery Unit (EDDU), Department of Neurology and Neurosurgery, Montreal Neurological Institute-Hospital, McGill University, 3801 University Street, Montreal, QC H3A 2B4 Canada; 2 Faculty of Medicine and Health Sciences, McGill University, 3605 De La Montagne, Montreal, QC H3G 2M1 Canada

**Keywords:** Amyotrophic lateral sclerosis, Denervation, Neuromuscular junction, TDP-43, Dying-back, Dying-forward

## Abstract

Amyotrophic lateral sclerosis (ALS) is a disease characterized by upper and lower motor neuron (MN) loss with a signature feature of cytoplasmic aggregates containing TDP-43, which are detected in nearly all patients. Mutations in the gene that encodes TDP-43 (*TARBDP*) are known to result in both familial and sporadic ALS. In ALS, disruption of neuromuscular junctions (NMJs) constitutes a critical event in disease pathogenesis, leading to denervation atrophy, motor impairments and disability. Morphological defects and impaired synaptic transmission at NMJs have been reported in several TDP-43 animal models and in vitro, linking TDP-43 dysregulation to the loss of NMJ integrity in ALS. Through the lens of the dying-back and dying-forward hypotheses of ALS, this review discusses the roles of TDP-43 related to synaptic function, with a focus on the potential molecular mechanisms occurring within MNs, skeletal muscles and glial cells that may contribute to NMJ disruption in ALS.

## Background

Amyotrophic lateral sclerosis (ALS) is an adult-onset degenerative disorder characterized by loss of upper and lower motor neurons (MNs) and progressive muscle atrophy [1]. Its prognosis is poor with symptoms progressing from weakness to fatal paralysis of respiratory function within two to four years after disease onset [2, 3]. Currently, there is no known cure and the few approved disease-modifying treatment options (i.e., riluzole [4–6], edaravone [7, 8] and the newly approved albrioza [9–11]) offer only modest benefits. About 10% of cases exhibit a Mendelian inheritance (termed familial ALS) and, to this day, > 30 genes have been associated with ALS through genetic studies [12]. The most commonly mutated genes include chromosome 9 open reading frame 72 (*C9ORF72*), superoxide dismutase 1 (*SOD1*), fused in sarcoma (*FUS*) and transactive-response DNA-binding protein (*TARDBP*) [13]. *TARDBP* encodes TDP-43, a ubiquitously expressed DNA/RNA-binding protein involved in multiple steps of RNA metabolism. Heterozygous missense mutations in *TARDBP* are found in 3% and 1.5% of familial and sporadic ALS cases, respectively [14–18]. Although these mutations occur in a small subset of patients, the significant role of TDP-43 as a causative factor in ALS has been highlighted by its identification as the main component of proteinaceous aggregates in post-mortem tissues of ALS patients [19–21]. Interestingly, TDP-43-containing aggregates are present in over 95% of ALS cases, including those without pathogenic mutations in *TARDBP* [19–21], suggesting convergent disease mechanisms.

A longstanding debate in ALS research is the primary site of disease onset, which opposes the “dying-back” and “dying-forward” hypotheses. The dying-back hypothesis posits that the disease process is initiated distally at the neuromuscular junction (NMJ) and progresses in a retrograde fashion to affect the axons and MN cell bodies. In support of this theory, studies have described early muscle denervation before the appearance of motor deficits in both patients and mouse models [22–25]. Analysis of muscle biopsies from ALS patients revealed abnormally small motor terminals and frequently denervated endplates, accompanied by electrophysiological evidence of presynaptic involvement [26]. However, this theory does not offer a clear explanation on how neurodegeneration of spinal MNs (lower MNs) may propagate to affect MNs of the motor cortex (upper MNs). In contrast, the “dying-forward” hypothesis proposes that the pathology has its origin in the motor cortex where dysfunctional upper MNs trigger the death of lower MNs via glutamate excitotoxicity, resulting in NMJ disruption and muscle atrophy. In line with this idea, studies have repeatedly reported early cortical hyperexcitability in ALS patients, sometimes preceding symptom onset [27–30]. Furthermore, chronic excitotoxic insults to lower MN soma have been shown to cause neurodegeneration, axonal fragmentation and NMJ retraction in mice [31]. A detailed overview of the evidence in support of both hypotheses is beyond the scope of this review; however, we direct the reader to several reviews on this topic [32–36].

Regardless of the primary site of neurodegeneration, the disruption of NMJs is a critical event in the pathogenesis of ALS, leading to denervation atrophy and weakness. Both loss-of-function and ALS mutations in *TARDBP* have been linked to axonopathy and NMJ pathology in several animal and cellular models (Table 1), further implicating TDP-43 as a key player in this disease. In this review, we describe the physiological and pathological roles of TDP-43 as they relate to synaptic maintenance and function, with an emphasis on TDP-43 dysregulation in MNs, skeletal muscles and glial cells as a potential driver of NMJ disruption in ALS. Further, we aim to discuss the proposed mechanisms from the perspectives of the dying-back and dying-forward hypotheses and provide suggestions for future investigations.

**Table 1:** Genetic models exploring association of TDP-43 with NMJ pathology

Animal model/study	TDP-43 expression	Observed phenotypes						
		NMJ defects	Impaired synaptic transmission at the NMJ^a^	Axonopathy	Muscle pathology	Motor deficits	Decreased lifespan	MN loss
**Fruit fly (Drosophila melanogaster)**								
Feiguin et al. [37], Godena et al. [39], Donde et al. [41]	TBPH KO (chromosomal deletion)	✓	na	na	**X**	✓	✓	**X**
Li et al. [38]	hTDP-43 OE in MNs	✓	na	✓	na	✓	✓	✓
Lin et al. [43]	TBPH KO (imprecise excision)	✓	na	✓	na	✓	✓	na
	TBPH OE in MNs	✓	na	✓	na	✓	✓	na
	TBPH KD in neurons	✓	na	na	na	✓	na	na
Wang et al. [40]	TBPH KO (chromosomal deletion)	**X**	na	na	na	✓	✓	na
	TBPH OE in MNs	✓	na	na	na	na	na	na
	hTDP-43 OE in MNs	✓	na	na	na	na	na	na
	hTDP-43 M337V OE in MNs	**X**	na	na	na	na	na	na
Diaper et al. [44]	TBPH KO (imprecise excision)	**X**	✓	na	**X**	✓	✓	✓
	TBPH OE (single inserts)	**X**	✓	na	na	✓	✓	✓
	TBPH KD in neurons (RNAi)	na	✓	na	na	✓	na	na
	TBPH KD in muscle (RNAi)	na	**X**	na	na	na	na	na
	TBPH KD in upper MNs (RNAi)	na	✓	✓	na	✓	**X**	✓
	TBPH OE in upper MNs	na	✓	✓	na	✓	**X**	✓
Estes et al. [42]	hTDP-43 WT OE in MNs	✓	na	✓	na	✓	✓	✓
	hTDP-43 A315T OE in MNs	**X**	na	X	na	✓	✓	✓
	hTDP-43 A315T OE (high expression) in MNs	✓	na	X	na	✓	✓	✓
Estes et al. [45]	hTDP-43 WT, D169G, G298S, A315T, N345K OE in neurons	✓	na	na	na	✓	na	na
	hTDP-43 WT, D169G, G298S, A315T, N345K OE in glia (pan-glial)	✓	na	na	na	✓	na	na
Coyne et al.[103, 203]	hTDP-43 WT, G298S OE in MNs	✓	✓	na	na	✓	✓	na
Romano et al. [46]	Inducible KD of TBPH in neurons (RNAi)	✓	na	na	na	✓	✓	na
	Inducible OE of TBPH in neurons	na	na	na	na	✓	na	na
Deshpande et al. [47]	TBPH KO (chromosomal deletion)	✓	na	na	na	na	✓	na
	hTDP-43 OE in MNs	✓	na	na	na	na	✓	na
Strah et al. [104]	TBPH KO (chromosomal deletion)	✓	✓	✓	na	✓	na	na
	TBPH KD in muscle (RNAi)	✓	na	na	**X**	✓	✓	na
Lee et al. [254]	hTDP-43 OE in glia (pan-glial)	✓	na	na	na	✓	✓	na
**Zebrafish (Danio rerio)**								
Kabashi et al. [48, 49], Campanari et al. [52]	hTDP-43 A315T, G348C, A382T OE	na	na	✓	na	✓	na	✓
	*tardbp* KD (AMO)	✓	na	✓	na	✓	na	**X**
Armstrong & Drapeau [53], Patten et al. [54]	hTDP-43 G348C OE	✓	✓	na	**X**	✓	na	✓
Dzieciolowska et al. [50]	*tardbpl* KD (AMO)	✓	**X**	na	na	**X**	**X**	na
	*tardbp* Y220X (unstable and degraded tdp-43)	✓	**X**	na	na	**X**	**X**	na
	*tardbp* Y220X + *tardbpl* KD	✓	✓	na	na	✓	✓	na
Bose et al. [51]	*tardbp* Y220X	✓	**X**	na	na	**X**	**X**	na
	*tardbpl* KO (5 bp deletion with CRISPR/Cas9)	✓	**X**	na	na	**X**	**X**	na
	*tardbp* Y220X and *tardbpl* KO (het/hom)	✓	✓	na	na	✓	**X**	na
	*tardbp* Y220X and *tardbpl* KO (hom/hom)	✓	✓	na	na	✓	✓	na
Asakawa et al. [85]	Optogenetic WT tdp-43	✓	na	✓	na	na	na	na
	Optogenetic WT hTDP-43	na	na	na	na	**X**	na	na
	Optogenetic hTDP-43 A315T	na	na	na	na	✓	na	na
**Rat (Rattus norvegicus)**								
Zhou et al. [56]	Inducible OE of hTDP-43 M337V (TET-off)	✓	na	✓	✓	✓	✓	✓
**Mouse (Mus musculus)**								
Shan et al. [133]	WT hTDP-43 OE	✓	na	✓	✓	✓	✓	**X**
Swarup et al. [59]	WT, A315T, G348C KI in *TARDBP* (BAC)	✓	na	✓	na	✓	na	**X**
Arnold et al. [60]	hTDP-43 Q331K OE	✓	✓	✓	✓	✓	na	✓
	hTDP-43 Q331K (low) OE	**X**	na	na	**X**	✓	na	✓ (trend)
	hTDP-43 M337V OE	**X**	na	na	**X**	✓	na	✓ (trend)
Mitchell et al. [61]	hTDP-43 Q331K OE	✓	na	X	✓	✓	**X**	✓ (trend)
	Coexpression of hTDP-43 WT and Q331K	✓	na	✓	✓	✓	✓	✓
Wegorzewska et al. [55], Marques et al. [188]	hTDP-43 A315T OE	✓	✓	✓	✓	✓	✓	✓
Walker et al. [117], Spiller et al. [118], Altman et al. [136]	Inducible hTDP-43ΔNLS (TET-off)	✓	✓	✓	✓	✓	✓	✓
Chand et al. [62]	hTDP-43 Q331K OE	✓	✓	✓	na	✓	na	✓
Wang et al. [256]	Selective cKO of *Tardbp* in oligodendrocytes	**X**	na	✓	na	✓	✓	**X**
White et al. [87]	Q331K-equivalent KI in *Tardbp* (CRISPR/Cas9)	**X**	**X**	na	**X**	**X**	na	**X**
Ebstein et al. [63]	M337V, G298S KI in *TARDBP* (BAC)	✓ (hom)	na	na	na	na	na	✓
Gordon et al. [64], Williamson et al. [65], Sleigh et al. [58]	M337V KI in *TARDBP* (BAC)	✓	na	✓	✓	✓	✓	**X**
White et al. [66]	hTDP-43 Q331K OE	✓	na	✓	✓	✓	na	✓
Huang et al. [57]	A315T, N390D-equivalent KI in *Tardbp* (BAC)	✓ (N390D)	na	✓ (N390D)	✓ (N390D)	✓ (N390D)	✓ (N390D)	✓ (N390D)
Peng et al. [255]	Selective cKO of *Tardbp* in astrocytes	**X**	na	na	na	✓	na	**X**
**Human cell-based models**								
Osaki et al. [67]	Patient-derived TDP-43 G298S iPSCs differentiated into MN spheroids and co-cultured with WT human iPSC-derived skeletal muscle	✓	✓	✓	✓	–	–	✓
Pereira et al. [68]	CRISPR/Cas9-edited TDP-43 G298S iPSCs differentiated into sensorimotor organoids	✓	na	**X**	**X**	–	–	**X**

✓ yes, X no, *na* not assessed, – not applicable, *AMO* antisense morpholino oligonucleotide, *BAC* bacterial artificial chromosome, *cKO* conditional knockout, *het* heterozygous,* hom* homozygous, *hTDP-43* human TDP-43, *iPSC* induced pluripotent stem cell, *KD* knockdown, *KI* knock-in, *KO* knockout, *LOF* loss-of-function, *MNs* motor neurons, *NLS* nuclear localization signal, *NMJ* neuromuscular junction, *OE* overexpression, *WT* wild type

^a^Impaired synaptic transmission at the NMJ was assessed using electrophysiological or optogenetic methods.

## Pathological dysregulation of TDP-43 is linked to NMJ disruption

Several reports using various TDP-43 models have linked TDP-43 dysfunction to NMJ abnormalities (Table 1). Earlier studies using loss-of-function or overexpression models have established that tightly regulated levels of TDP-43 are essential for normal NMJ development [37–40]. In *Drosophila*, both gain- and loss-of-function of TDP-43/TBPH cause morphological alterations at NMJs (e.g., abnormal axonal branching and changes in synaptic bouton number and shape), resulting in impairments of synaptic transmission, locomotive deficits and reduced lifespan [37–47]. Similarly, zebrafish lacking TDP-43 display aberrant motor axonal projections with reduced synaptic transmission at the NMJ and impaired locomotor function [48–52]. These efforts have led to the hypothesis that dysregulation of TDP-43 in ALS contributes to NMJ pathology, and motivated investigations on the effects of ALS-associated TDP-43 variants at this synapse. Similar to loss-of-function models, zebrafish expressing TDP-43 variants (A315T, A382T and G348C) show abnormal NMJ morphology and function along with swimming deficits [48, 49, 53, 54]. Several TDP-43 rodent models show early NMJ denervation and axonopathy that sometimes precede or coincide with the onset of motor deficits [55–66], consistent with a potential role of TDP-43 dysregulation in NMJ disruption.

Recently, the development of in vitro NMJ models using induced pluripotent stem cells (iPSCs) allowed exploration of the impact of ALS TDP-43 variants at this synapse in a human model. Patient-derived MN spheroids expressing TDP-43^G298S^ co-cultured with 3D skeletal muscle bundles form fewer thick neural fibers and NMJs compared to control motor units, resulting in reduced muscle contraction force [67]. Sensorimotor organoids derived from gene-edited TDP-43^G298S^ iPSCs exhibit a decreased area of innervated NMJs compared with isogenic controls [68]. Taken together, these studies present compelling evidence connecting TDP-43 to NMJ defects.

## Potential mechanisms underlying MN dysfunction and NMJ disruption

**Fig. 1:**
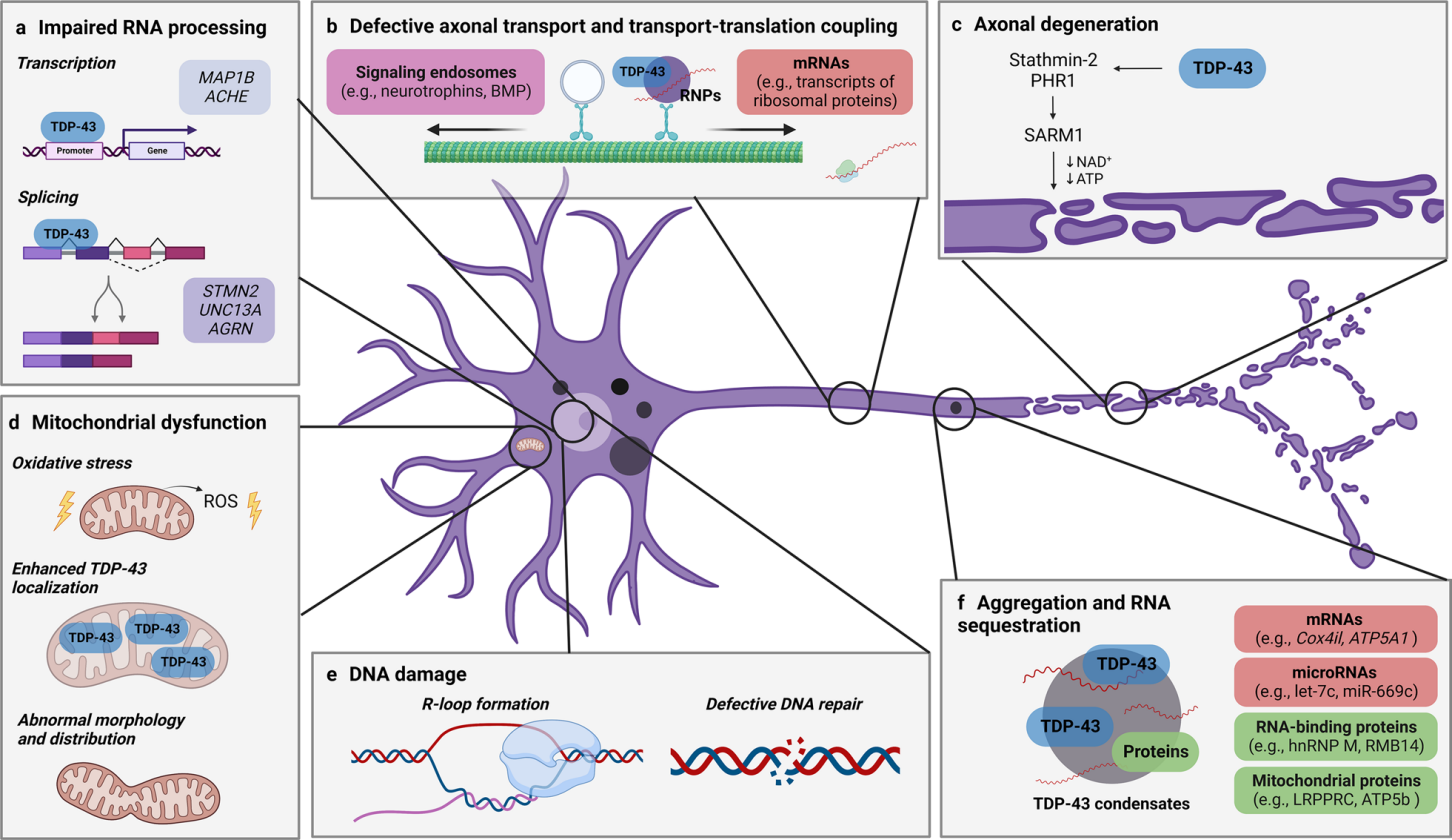
Potential mechanisms underlying MN dysfunction and NMJ disruption via dysregulated TDP-43. In the healthy cell, TDP-43 is involved in several key cellular functions including transcription, splicing, microRNA biogenesis, DNA repair, axonal transport, and translation. In the context of ALS, TDP-43 nuclear depletion, cytoplasmic mislocalization and aggregation may critically alter its functions, eventually leading to NMJ dismantling and MN loss. **a** Dysregulated TDP-43 may lead to synaptic destabilization through mis-splicing and/or altered expression of transcripts encoding proteins with critical roles at the NMJ. **b** Defective anterograde axonal transport of mRNAs along with impaired transport-translation coupling may impact local protein synthesis at presynaptic membranes, thereby compromising the integrity of NMJs. Impairments in retrograde axonal transport may disrupt the long-range signal transduction required to respond appropriately to external stimuli and maintain NMJ integrity and function. **c** Pathologically altered TDP-43 may confer increased susceptibility to activation of the Wallerian degeneration pathway, leading to axonal fragmentation and retraction of motor terminals. **d** Oxidative stress, enhanced mitochondrial localization of TDP-43 along with abnormal mitochondrial morphology and distribution may induce the loss of MNs and NMJs. **e** Failure of DNA repair mechanisms mediated by TDP-43 may trigger distal axonal defects and NMJ dismantling. **f** TDP-43 condensates may sequester mRNAs, microRNAs and proteins, thereby depleting MNs of key factors for NMJ maintenance

In healthy cells, TDP-43 is predominantly localized in the nucleus where it regulates multiple steps of gene expression including transcription [69] and splicing [70] and participates in DNA repair [71, 72]. In addition, a small proportion of the protein is localized in the cytoplasm where it is involved in mRNA stabilization and transport [73–76], translation [77, 78], microRNA biogenesis [79, 80] and stress granule assembly [81–84]. In the context of ALS, TDP-43 becomes depleted from the nucleus and mislocalizes in the cytoplasm where it accumulates and forms insoluble aggregates [19–21]. These changes in subcellular localization and solubility may critically alter the functions of TDP-43 (most probably via a combination of loss- and gain-of-function mechanisms), which eventually exerts deleterious effects on NMJs and MN survival. In the translucent zebrafish, optogenetic induction of cytoplasmic mislocalization and aggregation of wild-type TDP-43 is sufficient to trigger axonal defects and endplate denervation [85], consistent with the hypothesis that pathogenic dysregulation of TDP-43 may underlie NMJ disruption. We focus our attention on perturbed TDP-43 functions of potential importance for the loss of NMJ integrity in ALS (Fig. 1).

### Impaired RNA processing

In ALS, it is hypothesized that the loss of nuclear localization of TDP-43 may alter RNA processing that usually occurs in the nucleus, which may lead to dysfunction of cellular pathways critical for neuron health and NMJ integrity. In fact, mutations in *TARDBP* have been shown to cause various RNA abnormalities such as changes in gene expression, mis-splicing and reduced transcript stability [60, 86–89]. While TDP-43 normally functions as a splicing repressor regulating the inclusion of alternatively spliced exons [41], widespread splicing alterations have repeatedly been described in TDP-43 downregulation and mutant models [60, 87–94]. Pathologically altered TDP-43 can induce (1) the inclusion of normally excluded exons (cryptic exons) [91–94], and (2) the exclusion of normally constitutively expressed exons (skiptic exons) [88], suggesting both loss- and gain-of-function mechanisms with regard to TDP-43 splicing functions [88]. Incorrect splicing can cause a frameshift, introduction of a stop codon and/or generation of an aberrant splicing product that yields a non-functional protein. Of note, splicing alterations have sometimes been observed without detectable aggregation or nuclear clearing [60] and in the absence of neurodegeneration [89], implying that impaired RNA processing may be an early event in ALS pathogenesis.

The first studies characterizing RNA targets of TDP-43 using cross-linking immunoprecipitation combined with high-throughput RNA sequencing revealed that TDP-43 binds to thousands of transcripts derived from genes implicated in RNA metabolism, neurodevelopment, neuronal survival and synaptic function [90, 95–97]. Polymenidou and colleagues found that the most downregulated genes in TDP-43-depleted mouse brains encode proteins critical for synaptic formation and neurotransmission such as glutamate receptor subunits (*Gria2/3*, *Grik2*, *Grin1*, *Grin2a/b*), ion channels (*Cacna1*, *Kcnma1*) and synaptic vesicle proteins neurexin 1 to 3 (*Nrxn1/2/3*) and neuroligin 1 (*Nlgn1*) [90]. Similarly, analysis of post-mortem cortical tissues of patients with TDP-43 pathology revealed significant downregulation of genes involved in synaptic functions, including synaptic vesicle proteins synaptobrevin 1 (*VAMP1*), synaptotagmins (*SYT1*, *SYT13*) and synaptosomal-associated protein 25 (*SNAP25*) [98]. Recently, loss of TDP-43 was found to induce cryptic splicing of the critical synaptic gene *UNC13A* in iPSC-derived motor and cortical neurons and post-mortem brain neuronal nuclei, resulting in depletion of the *UNC13A* transcript and protein [94, 99]. Furthermore, single nucleotide polymorphisms (SNPs) in *UNC13A* (associated with increased ALS and frontotemporal dementia (FTD) risk through genome-wide association studies) were found to promote this incorrect splicing in patient brain tissues [94].

Differential expression of synaptic transcripts was also observed in several cellular and animal TDP-43 models [46, 60, 100]. Of particular interest, some studies revealed interactions between TDP-43 and transcripts encoding proteins with critical roles at the neuromuscular synapse [52, 95]. TDP-43 binds the *AGRN* transcript encoding agrin [95], a key regulator of NMJ development and maintenance [101]. *AGRN* was shown to undergo cryptic splicing upon TDP-43 depletion [93, 94]. Lower levels of agrin are detected in the cerebrospinal fluid of ALS patients compared with non-ALS patients and healthy controls [102]. Additionally, TDP-43 was found to directly interact with the *MAP1B* transcript [95], which encodes a protein responsible for stabilizing microtubules at presynaptic terminals during NMJ formation. Altered subcellular localization of *MAP1B* transcripts has been described in spinal cord specimens of ALS patients [103]. Levels of the *MAP1B* ortholog *futsch* have been repeatedly shown to be decreased with TBPH/TDP-43 loss-of-function in flies [39, 46, 104]. Interestingly, mutations in *futsch* phenocopy several pathogenic changes observed with TBPH/TDP-43 depletion [105], supporting the idea that TDP-43 dysfunction may result in structural defects at the NMJ. Recently, a novel role of TDP-43 in regulating acetylcholinesterase (AChE) expression was described [52]. AChE, classically known for hydrolyzing the neurotransmitter acetylcholine (ACh) in the synaptic cleft, has been demonstrated to be involved in NMJ development and NMJ stabilization at the adult synapse [106–109]. TDP-43 knockdown in zebrafish is associated with decreases of AChE activity and expression, while overexpression of human AChE ameliorates NMJ pathology and locomotive deficits [52]. Moreover, reduced transcript levels of *ACHE* have been reported in ALS spinal cord tissue sections related to the site of symptom onset [110], highlighting a potential contribution of AChE to disease pathogenesis.

Overall, these findings strengthen the hypothesis that dysregulated TDP-43 may lead to synaptic destabilization through altered gene expression. Given the thousands of RNA targets regulated by TDP-43, the challenge now is to identify the transcriptomic changes most relevant to the development and progression of ALS.

### DNA damage

In addition to impaired RNA processing, TDP-43 dysfunction has been linked to defective DNA damage response (DDR) [71, 72]. In healthy neurons, TDP-43 is involved in the detection and repair of double-stranded DNA breaks (DSBs) via non-homologous end joining (NEHJ) [71, 72], a major DNA repair pathway as neurons are unable to divide or undergo homologous recombination. TDP-43 is rapidly recruited at DNA damage sites where it interacts with factors of DDR and NHEJ-mediated DSB repair, including the XRCC4–DNA ligase 4 complex [71, 72, 111]. TDP-43 depletion in multiple neuronal cell models causes a significant accumulation of DSBs due to a reduction in NHEJ-mediated DSB repair efficiency [71, 72]. In particular, TDP-43 is involved in the prevention and repair of transcription-associated DNA damage, specifically, the formation of R-loops [112, 113]. These are three-stranded DNA:RNA hybrid structures which can lead to spontaneous DSBs when unresolved. In HeLa cells, silencing of TDP-43 leads to increased R-loop formation and R-loop-mediated DNA damage [113].

Hence, it is hypothesized that the loss of TDP-43 nuclear functions in ALS may cause persistent DNA repair defects and genome instability. In fact, TDP-43 nuclear clearing correlates with DNA damage and activation of DDR in sporadic ALS spinal cord tissues [71]. Similarly, transfection of TDP-43^A315T^ and TDP-43^Q331K^ in multiple cellular models lead to higher levels of the DSB marker γH2AX, indicating a loss of DNA repair function induced by ALS mutations [72, 114]. Increased DNA damage was detected in spinal cord tissues from patients expressing TDP-43^Q331K^ [114] as well as in the frontal cortex of patients with FTD-TDP-43 [115]. Interestingly, fibroblasts obtained from two pre-symptomatic individuals with *TARDBP* mutations encoding TDP-43^M337V^ also display increased levels of DNA damage and impaired NHEJ, implying that failure of DNA repair mechanisms by TDP-43 may occur early in the disease course [72].

Focussing here on potential mechanisms of NMJ disruption, it could be hypothesized that persistent DNA damage can provoke MN death [116], thereby triggering the retraction of motor terminals. An alternative hypothesis is that DNA damage in MNs may cause NMJ dismantling prior to neurodegeneration. Consistent with this idea, early accumulation of DNA damage was detected in the cortex of inducible hTDP-43ΔNLS mice preceding NMJ denervation, followed later by spinal MN loss [72, 117, 118]. Although this study did not examine the presence of DNA damage in spinal cord tissues, another group established a link between early DNA damage and distal axonal defects [119]. Naumann and colleagues performed a sequential characterization of mutant *FUS* phenotypes in iPSC-derived MNs and reported early DNA damage, followed by defects in axonal trafficking of organelles, axonal degeneration, and finally death of MNs [119]. Unfortunately, to our knowledge, no equivalent study has yet been performed in a TDP-43 model. Overall, these studies support a critical role of defective DNA repair mechanisms by dysfunctional TDP-43 in the pathogenesis of ALS. Further work is required to determine the downstream consequences of DNA damage and how they may relate to denervation.

### Mitochondrial dysfunction

Mitochondria are the main producers of reactive oxygen species (ROS), which cause oxidative stress and lead to cell death through apoptosis at excessive amounts [120]. Mitochondria also play a critical role in energy production, which is crucial for MNs due to their high metabolic demand to sustain their large size and long axons. Oxidative stress and metabolic imbalance can result from mitochondrial dysfunction, which is hypothesized to contribute to ALS pathogenesis. In fact, evidence of increased oxidative stress was found in the motor cortex [121, 122] and spinal cord [123] of sporadic ALS patients. Additionally, abnormal mitochondrial morphology was observed in ALS spinal cord specimens [121].

Mitochondrial dysfunction has been repeatedly described in cellular models expressing human wild-type TDP-43 or ALS variants (Q331K, M337V, A382T, I383T), including increased levels of mitochondrial ROS [124], activation of mitophagy [125, 126], reduced basal respiration [127] and transmembrane potential [128], and deficiency in calcium uptake [129]. While TDP-43 is normally detected in mitochondria, this localization is increased in ALS patient specimens [130]. TDP-43 mitochondrial localization is also enhanced by TDP-43 variants [131, 132], perhaps reflecting a gain of toxic function. Consistent with this idea, inhibition of TDP-43 mitochondrial localization mitigates neurodegeneration and NMJ loss in TDP-43^A315T^ mice [132].

Mitochondria have also been detected within large TDP-43 aggregates in TDP-43 transgenic mice [133, 134], leading to the hypothesis that aggregates may sequester this organelle. Furthermore, aggregates have been shown to dysregulate the expression of nucleus-encoded mitochondrial proteins via sequestration of mRNA, microRNAs and other RNA-binding proteins, resulting in enhanced oxidative stress [135], fewer and dysfunctional mitochondria at NMJ pre-synapses, and denervation [136].

Abnormalities in mitochondrial morphology and distribution are a prominent TDP-43 phenotype [126, 127, 131, 134, 136–138]. Furthermore, abnormal mitochondria have been shown to accumulate in presynaptic terminals of ALS patients [121], although this has been recapitulated inconsistently in TDP-43 transgenic mice. Two studies have described depletion of mitochondria at nerve terminals of NMJs in mice expressing human wild-type TDP-43 [133] or hTDP-43**Δ**NLS [136]. In concordance with post-mortem studies, Magrané and colleagues noted an accumulation of mitochondria in distal axons and at NMJs of presymptomatic mice expressing TDP-43^A315T^ [138]. Despite these conflicting results, both accumulation and depletion of mitochondria may have profound consequences at the NMJ, as the localization and integrity of mitochondria at nerve terminals is directly correlated with NMJ function [136, 139, 140].

In summary, mitochondrial dysfunction is commonly linked to TDP-43 dysregulation. In ALS, aggregation and enhanced mitochondrial localization of TDP-43 along with abnormal distribution of mitochondria may induce the loss of MNs and NMJs.

### Defective anterograde axonal transport and transport-translation coupling

In the cytoplasm, TDP-43 associates with RNA and other effector proteins to form transport ribonucleoproteins (RNPs) responsible for RNA transport along microtubules in both anterograde and retrograde trajectories [73–76]. This enables control of protein expression in specific regions of the cell, a process that is particularly important for MNs as they are large cells with multiple cellular compartments (cell body, dendrites and axons) that have local translational needs. Altered axonal transport has been one of the earliest proposed mechanisms to explain NMJ disruption in ALS and constitutes a frequently identified phenotype in TDP-43 models [74, 138, 141]. Furthermore, genetic defects and abnormalities in cytoskeletal components and motor complexes are commonly linked to ALS [142–146] (reviewed in [147]).

One hypothesis is that impairment in anterograde transport (from the cell body to neuronal processes) may prevent adequate maintenance of distal axons and presynaptic membranes, leading to denervation and neuronal cell death. ALS-associated mutations in *TARDBP* (M337V, A315T and G298S) have been shown to decrease anterograde transport and enhance accumulation of transport RNPs in the cell body [74]. As a result, delivery of transcripts to distal compartments is impaired, as shown by altered mRNA content in axonal processes of mutant MNs [74]. Similarly, axon sequencing (axon-seq) analyses identified broad changes in the subcellular localization of mRNAs and microRNAs in the cell soma and axons of primary mouse MNs depleted of TDP-43 or expressing the TDP-43^A315T^ variant [148, 149]. Thus, it is conceivable that alterations in the spatiotemporal localization of RNA species within MNs due to defective axonal transport may impact local protein synthesis at the presynaptic membrane, compromising the integrity of neuromuscular synapses.

TDP-43 is detected at presynaptic membranes of NMJs [74, 150], suggesting that it may also directly contribute to local regulation of translation at this synapse. At least in dendrites, there is accumulating evidence that TDP-43 regulates local translation along with Fragile X mental retardation protein (FMRP) [73, 77, 78, 151, 152]. TDP-43 acts as a translational repressor and stabilizes RNA until a stimulus (such as neuronal activity) signals a need for novel proteins at the synapse [78]. Given that TDP-43 interacts with the D1 domain of FRMP via its C-terminal domain (where the vast majority of ALS mutations cluster) [151], it has been proposed that this interaction could be perturbed in ALS, preventing MNs from adequately modulating transport-translation coupling of RNPs [73]. Interestingly, loss-of-function mutations in the FMRP ortholog dFXR lead to morphological defects and alterations of neurotransmission at the NMJ in fruit flies [153, 154].

Moreover, Nagano and colleagues recently showed that TDP-43 binds and transports along axons the mRNAs of ribosomal proteins (RPs) that are locally translated and assembled into ribosomes which, in turn, participate in local protein synthesis themselves [155]. Using in situ hybridization, they showed that the RP mRNA signal is significantly decreased along axons of TDP-43-depleted mouse cortical neurons [155], revealing a broader role of TDP-43 in modulation of protein synthesis. It is worthy noting that, in addition to RNPs, the delivery of other vital cargos which depends on anterograde transport to reach the pre-synaptic compartment (e.g., synaptic vesicles precursors, mitochondria and proteins [139, 140, 156, 157]) may also become compromised in TDP-43-ALS.

### Defective retrograde axonal transport

Another proposed mechanism for NMJ disruption in ALS is the abnormalities of retrograde transport that may prevent the delivery of factors supporting neuron survival back to the cell body, such as neurotrophin-containing signaling endosomes [23]. Neurotrophins (such as brain-derived neurotrophic factor and nerve growth factor) are normally internalized through receptor-mediated endocytosis and retrogradely transported to cell bodies to modulate various aspects of the developing and adult neurons including cell survival, neurite outgrowth and synaptic function [158]. The TDP-43^M337V^ variant was recently found to impair the retrograde axonal transport of neurotrophin-containing signaling endosomes in mice, preceding NMJ dismantling and motor symptoms [58].

In addition to neurotrophins, other pathways that initiate at the NMJ are crucial for regulation of the formation and function of this synapse, including the bone morphogenetic protein (BMP) signaling pathway [159, 160]. Mutations in essential components of this signaling cascade (i.e., BMP, BMP receptors and Smad transcription factors) induce changes in NMJ morphology and a decrease in neurotransmitter release [159, 160]. In fruit flies, defects in endocytic traffic of BMP receptors have been described with both loss- and gain-of-function of TDP-43/TBPH, as demonstrated by a shift from Rab5^+^ early endosomes to Rab11^+^ recycling endosomes at motor terminals [47]. These results were accompanied by a decrease in pMAD staining indicative of decreased BMP signaling at the NMJ, while rerouting BMP receptors via Rab11 inhibition partially restores BMP signaling, NMJ defects and motor deficits [47]. There is also pathological evidence of dysfunctional BMP/TGF-β signaling in sporadic ALS spinal cord specimens, with MNs showing accumulation of pSmad in cytosolic TDP-43 aggregates [161]. Taken together, TDP-43 dysfunction could prevent MNs from maintaining the integrity of NMJs by disrupting the long-range signal transduction required to respond appropriately to external stimuli.

### Axonal degeneration

Axonal fragmentation is a prominent feature of neurodegeneration. According to the dying-back theory, degeneration originates distally at nerve terminals and progresses in a retrograde fashion to sequentially affect the axons and cell bodies, eventually leading to MN loss [23, 25]. This phenomenon is reminiscent of Wallerian degeneration (also known as programmed axon death), a tightly regulated process of axonal fragmentation and neuronal death, distinct from apoptosis, which occurs following a nerve injury [162, 163]. Sterile Alpha and TIR Motif-Containing 1 (*SARM1*) has been identified as a key initiator of programmed axon death, as depletion of this gene confers long-term resistance to degeneration [164–169]. The *SARM1* locus has been associated with an increased susceptibility to sporadic ALS [170] and constitutively active SARM1 variants have been recently identified in ALS patients [171, 172]. ALS, as well as other neurodegenerative diseases where axons may be affected before neuronal cells bodies (e.g., Parkinson’s disease, Alzheimer’s disease and Huntington’s disease [173–177]), is increasingly believed to be Wallerian-like disorders in which a similar cell death program is triggered in the absence of a physical insult. Metabolic stress and disruption of axonal transport, two processes which have been repeatedly associated with ALS pathophysiology, are thought to be responsible for initiating this response [146, 178–180]. In particular, studies have consistently reported both mitochondrial and axonal dysfunction in TDP-43 models [58, 74, 125, 129, 131, 138, 141], raising a possible link between TDP-43 and programmed axon death. The role of TDP-43 in response to cellular injury reinforces this hypothesis, as in vivo axotomy or axon ligation triggers upregulation and transient accumulation of TDP-43 at the site of injury [181–183]. Furthermore, TDP-43^G348C^ mice exhibit sustained cytoplasmic mislocalization of TDP-43 and impaired recovery after nerve crush injury, as shown by fewer regenerating axons and persistent motility impairments compared with control animals [184].

More direct evidence implicating TDP-43 in the Wallerian pathway was demonstrated by genetic ablation of *SARM1* resulting in improvement of disease phenotypes in *TARDBP* models [66, 185]. In *C. elegans* expressing TDP-43^A315T^, loss-of-function mutation in the *SARM1* ortholog *tir-1* improves motility deficits and MN survival [185]. Similarly, *SARM1* knockout mitigates axonal degeneration and MN loss in TDP-43^Q331K^ mice [66]. Importantly, these findings were accompanied by a significant decrease in NMJ denervation [66]. These results imply that the activation of the axonal death program is involved in disruption of NMJs, and preserving the motor terminal-muscle interaction and axonal integrity may be required for the survival of MN cell bodies [66]. Recently, patient-associated SARM1 variants were shown to promote neurodegeneration in primary neurons and mice, due to a constitutive NAD^+^ hydrolase activity [171, 172]. In this regard, we speculate that TDP-43 dysregulation in ALS may confer an increased susceptibility to activation of the Wallerian pathway via SARM1, causing NAD^+^ depletion (and consequently ATP depletion), axonal degeneration, NMJ denervation and MN loss. It is worthy of note, however, that *SARM1* deletion does not mitigate neurodegenerative phenotypes in the SOD1^G93A^ mouse model, suggesting distinct mechanisms in SOD1-ALS [186, 187].

TDP-43 is also associated with other mediators of the Wallerian pathway, namely PHR1 (also known as PLEKHB1) [188] and stathmin-2 (also known as SCG10) [189, 190]. PHR1 promotes Wallerian degeneration, as its conditional knockout delays degeneration of severed axons and NMJ loss similar to *SARM1* depletion [191]. Paradoxically, it is also involved in axon outgrowth and synaptic formation [192–194]. PHR1 is essential for the development of NMJs: its constitutive knockout is lethal at birth due to incomplete innervation of the diaphragm, causing respiratory failure [192, 194]. PHR1 is significantly downregulated in MNs of TDP-43^A315T^ mice in the early symptomatic phase of the disease, preceding NMJ morphological defects [188]. Further investigation is required to determine the possible pathological role of PHR1 in TDP-43-mediated ALS.

Two studies have clearly shown that TDP-43 regulates expression of stathmin-2 (*STMN2*) [189, 190], an axon-maintenance factor that is rapidly depleted in distal axons upon injury [195, 196]. It is considered an early marker of subsequent axonal degeneration, potentially acting upstream of *SARM1* [195]. Stathmin-2 was shown to be significantly downregulated in spinal cord and cortical specimens from ALS patients as well as in iPSC-derived MNs depleted of TDP-43 [189, 190]. Mechanistically, the decline of stathmin-2 level is due to altered TDP-43 splicing activity, causing the inclusion of a cryptic exon that results in a non-functional protein [189, 190]. Stathmin-2 downregulation has also been observed in patient-derived neurons expressing TDP-43 variants (G298S, A382T, N390S), suggesting a loss of normal splicing function (i.e., cryptic exon repression) conferred by the mutations [190]. Loss of stathmin-2 is associated with impaired axonal regeneration following in vitro axotomy [189, 190], consistent with its role in maintaining the integrity of axons. Stathmin is also shown to be required for maintenance of NMJ stability. In fruit flies, neuron-specific knockdown of *stathmin*, or expression of a loss-of-function mutant, causes a reduction of bouton number and axonal retractions at the NMJ [197, 198]. Similarly, *Stathmin* mutant or knockout mice develop a late-onset axonopathy and NMJ denervation, leading to muscle atrophy and severe motor impairments [199, 200].

In summary, TDP-43 is functionally linked to factors involved in the Wallerian degeneration pathway, with dual roles in axonal outgrowth and NMJ maintenance. Disturbances in TDP-43 homeostasis in ALS may affect the expression levels of these factors, which in turn may contribute to defects at the NMJ, axonal degeneration and MN loss that characterize this disease.

### Aggregation and RNA sequestration

TDP-43 aggregation is a core feature of ALS [20]. These insoluble aggregates, detected in nearly all ALS cases, contain ubiquitinated and hyperphosphorylated full-length TDP-43 as well as truncated C-terminal fragments of the protein [20]. When ALS-associated mutations are present in *TARDBP*, TDP-43 has an increased propensity to aggregate and is capable of interacting with the wild-type protein, recruiting it into further aggregates [61, 201]. The majority of mutations are found in exon 6 of *TARDBP* encoding the protein’s glycine-rich C-terminal domain, which has been proposed to mediate solubility and oligomerization [202]. This implies that aggregation may be an important contributor to disease phenotype.

It has been proposed that aggregates can sequester RNA from the translational machinery, thereby depleting MNs of critical proteins for NMJ maintenance. Indeed, an emerging property of pathologically altered TDP-43 is sequestration of mRNA into insoluble complexes [136, 203]. Coyne and colleagues have shown that the TDP-43^G298S^ variant can sequester transcripts of the chaperone Hsc-70-4/HSPA8, resulting in decreased expression of the protein at the NMJ in transgenic *Drosophila* and mice [203]. These changes are accompanied by deficits in synaptic vesicle endocytosis, defects in NMJs and locomotion, and decreased lifespan [203]. HSPA8 protein levels, but not transcript levels, are also reduced in human MNs differentiated from iPSCs expressing *C9ORF72* or *TARDBP* mutations, confirming a post-transcriptional mechanism of expression inhibition [203]. It is plausible that this process may take place within aggregates given their resemblance to RNA granules, which are known to contain mRNA in a translationally silent state (i.e., stalled translation initiation complexes) [204]. Under physiological conditions, TDP-43 participates in the assembly of both transport RNPs [73–76] and stress granules [81–84], highlighting its role in modulating mRNA availability in time and space. In ALS, a gain-of-function of TDP-43 could result in mRNA trapping within insoluble aggregates rather than being stabilized temporarily within granules. In support of this idea, Altman and colleagues showed that “aggregate-like” TDP-43 RNP condensates drive suppression of local protein synthesis in sciatic MN axons and presynaptic terminals of inducible hTDP-43ΔNLS mice [117, 118, 136]. Specifically, they demonstrated that mRNAs of nucleus-encoded mitochondrial genes *Cox4il* and *ATP5A1* are directly bound by TDP-43 and sequestrated within axonal condensates, resulting in decreased levels of the proteins [136]. Ceasing hTDP-43ΔNLS expression induces clearance of axonal and synaptic condensates and consequently restores local protein synthesis as well as the number of innervated NMJs and contracting muscle fibers. However, the precise mechanisms through which TDP-43 condensates inhibit protein synthesis remain to be investigated. In addition to mRNAs, Zuo and colleagues showed that H_2_O_2_-induced TDP-43 aggregates also sequester specific microRNAs in mouse neuroblastoma-derived N2a cells, leading to upregulation of their corresponding targets [135]. RNA immunoprecipitation experiments showed that TDP-43^M337V^ enhances the capture of the microRNAs compared with the wild-type protein, supporting a gain-of-function mechanism. Furthermore, TDP-43 co-aggregates with other RNA-binding proteins (i.e., hnRNP M, hnRNP H1 and RMB14), raising the possibility that RNA sequestration within aggregates may not be limited to direct TDP-43 targets. Taken together, these studies support the hypothesis that TDP-43 aggregates may negatively impact NMJs by interfering with the expression of essential proteins for NMJ maintenance and function.

## TDP-43 dysregulation in non-neuronal cell types

While MN dysfunction and degeneration has traditionally been the focus of ALS research, a growing body of evidence recognizes non-cell autonomous mechanisms exerted by cells interacting with MN cell bodies or presynaptic terminals. Here, we describe some of the studies focused on TDP-43 dysregulation in non-neuronal cell types which have been hypothesized to influence NMJ integrity in ALS.

### Skeletal muscle

**Fig. 2:**
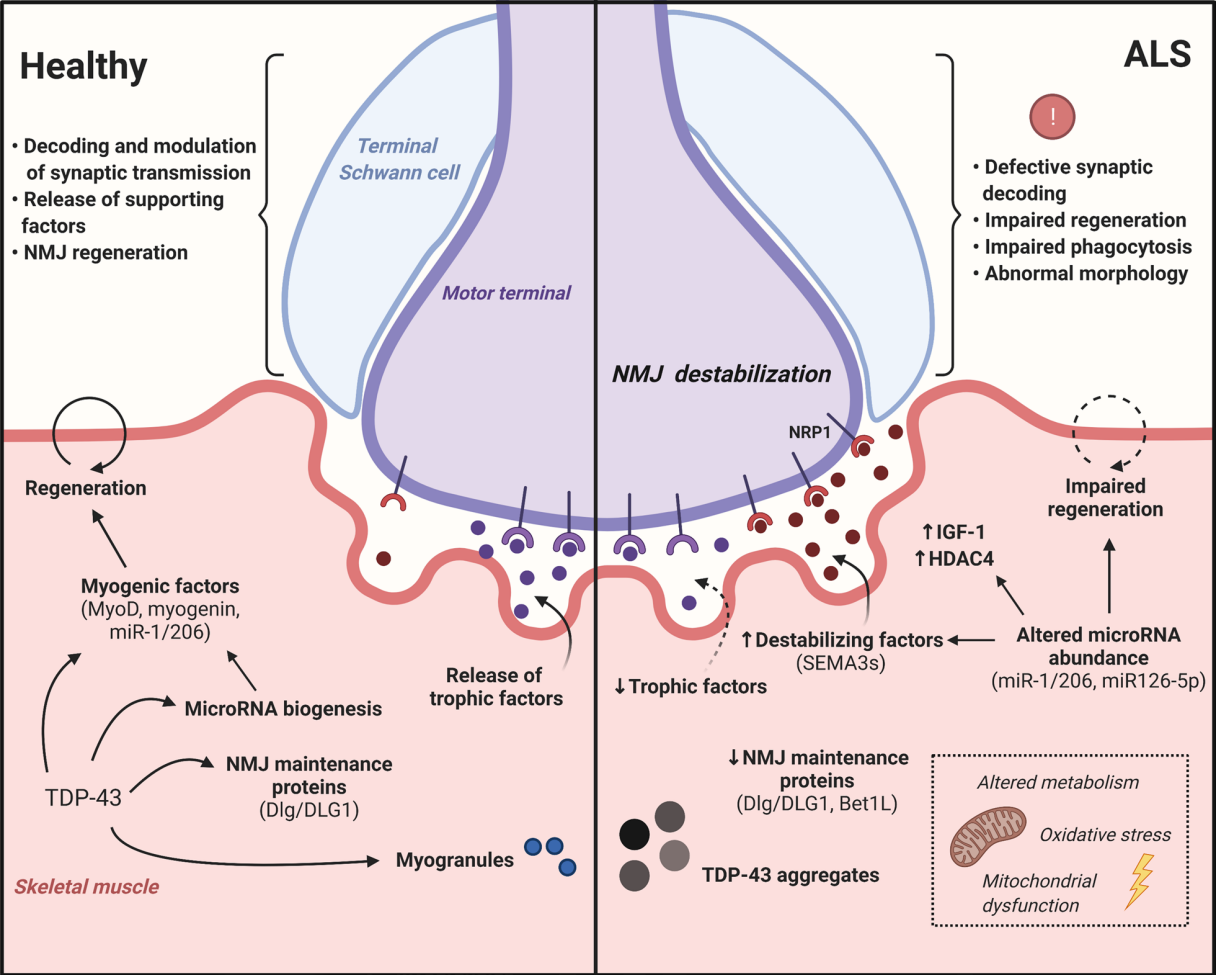
Dysfunction of non-neuronal components of the tripartite synapse may impact NMJ integrity in ALS. In the healthy skeletal muscle (left), TDP-43 promotes muscle regeneration and NMJ formation. In ALS (right), TDP-43 dysregulation impairs the expression genes encoding NMJ maintenance proteins (e.g., Dlg, Bet1L) as well as key microRNAs (e.g., miR-1, miR-206, miR2826-p), which may lead to denervation via the disorganization of presynaptic membranes and the release of destabilizing factors (e.g., SEMA3s). In addition to MN presynaptic terminals and muscle endplates, TSCs are the third cellular component of the tripartite synapse. TSC dysfunction (e.g., impairment in synaptic decoding and morphological abnormalities) may impact the ability to maintain NMJ integrity and promote reinnervation

The hypothesis that skeletal muscle may play an active role in disease initiation and progression emerged from studies describing muscle-specific pathogenic changes in human ALS specimens, such as mitochondrial morphology defects [205–207] and altered muscle oxidative metabolism [208–211]. In addition, studies performed with muscle tissue from ALS patients have reported abnormalities in factors secreted by skeletal muscle that could result in NMJ destabilization, including impaired expression of neurotrophic factors [212, 213] and increased levels of axon chemorepellent molecules [214–216]. Evidence of muscle dysfunction in ALS and identification of TDP-43 aggregates as the pathological hallmark of this disease have prompted further research into the physiological and pathological roles of TDP-43 in skeletal muscle (Fig. 2). TDP-43 aggregates have been detected in the skeletal muscle of patients with various myopathies including inclusion bodies myositis (IBM) [217, 218], the most common ALS-mimicking disease [219]. In addition, TDP-43 aggregates have been described in the muscle of patients with sporadic and familial ALS, with pathology associated with myogenic degeneration [220–222]. Indeed, the aggregates are identified predominantly in muscle fibers showing single-fiber atrophy as well as moderate to marked vacuolar degeneration [220].

TDP-43 is essential for muscle regeneration [223]. Vogler and colleagues showed that TDP-43 assembles into myo-granules containing sarcomeric transcripts, which may help coordinate sarcomere assembly during muscle formation [223]. They hypothesize that these myo-granules could be the precursors of pathogenic aggregates, forming in conditions of increased assembly or decreased clearance [223]. During muscle formation, TDP-43 is upregulated and binds to promoters of genes encoding important mediators of myogenesis (e.g., *MyoD*, *MYOG* and *Acta1*) to regulate their expression and initiate the differentiation program [224]. Militello and colleagues demonstrated that TDP-43 knockdown inhibits myogenic differentiation of C2C12 myoblasts, as shown by a decrease in the number of multi-nucleated myotubes [224]. In zebrafish, fruit flies and mice, both gain and loss of TDP-43 exert deleterious effects on skeletal muscle [225–227]. These findings raise the hypothesis that TDP-43 dysregulation in ALS may affect muscle regeneration. This idea is consistent with the reported impaired regenerative capacity of skeletal muscle in ALS, as satellite cells isolated from patients and presymptomatic animals are less proliferative and incapable of restoring mature myofibers [228, 229]. It is thus conceivable that muscle dysfunction could in turn negatively affect NMJ integrity.

TDP-43 expression in muscle has been shown to promote NMJ assembly [104]. In fruit flies, selective downregulation of TDP-43/TBPH in skeletal muscle is sufficient to induce locomotive and synaptic defects, with disorganization of both pre- and post-synaptic membranes [104]. Mechanistically, TDP-43/TBPH was shown to regulate levels of Discs-large (Dlg) [104], a protein expressed in both MNs and skeletal muscle that promotes NMJ formation by recruiting adhesion and scaffolding molecules [230–232]. Thus, TDP-43 dysregulation in muscle could prevent adequate expression of NMJ maintenance proteins such as Dlg, eventually resulting in NMJ dismantlement. Another study has implicated that altered gene expression within the muscle participates in disruption of NMJ architecture in ALS [233]. A transcriptome analysis identified *BET1L* as a commonly downregulated gene in iPSC-derived myocytes from *TARDBP*, *SOD1*, *C9ORF72* and sporadic ALS patients [233]. In the same study, they showed that Bet1L protein is localized at the basal lamina of the NMJ and that its expression levels decrease with disease progression in symptomatic SOD1^G93A^ rats [233].

Other groups have proposed that misregulation of non-coding RNAs in skeletal muscle may promote NMJ disruption. King and colleagues showed that TDP-43 negatively regulates the activity of muscle-enriched microRNAs of the miR-1 family (namely miR-1 and miR-206) [234] with functional roles in both differentiation of muscle progenitors and NMJ maintenance [235–238]. For instance, in *C. elegans*, miR-1 regulates the expression of acetylcholine receptor subunits and modulates ACh release from motor terminals via retrograde signaling [237]. MiR-206 is required for regeneration of NMJs after acute nerve injury in ALS mice [238]. TDP-43 inhibits the activity of miR-1/206 by preventing their association with the RISC complex, resulting in increased protein levels of their targets: insulin-like growth factor 1 (IGF-1) and histone deacetylase 4 (HDAC4) [234]. While high serum levels of IGF-1 are recently linked to a better disease prognosis in an ALS cohort [239], HDAC4 was reported to be upregulated in ALS muscle samples with levels correlating with disease progression and denervation [240, 241]. In addition, HDAC4 has been shown to inhibit muscle reinnervation in mice [238].

Co-culture of ALS myocytes with control MNs has provided additional evidence for a potentially toxic role of skeletal muscle via dysregulated TDP-43 [242, 243]. Wächter and colleagues found that control MNs exhibit impaired survival and decreased neurite length when co-cultured with mouse embryonic stem cell (mESC)-derived muscle expressing TDP-43^A315T^ [242]. Similarly, Maimon and colleagues reported a delay in the outgrowth of wild-type axons towards mouse primary myocytes transfected with TDP-43^A315T^ and several other ALS-linked mutations [243]. They also showed that muscle toxicity could be partially explained by dysregulation of microRNA miR126-5p [243], which was previously found to be downregulated in axons of primary MN cultures from both TDP-43^A315T^ and SOD1^G93A^ mouse models [148]. Depletion of miR126-5p caused an increase in levels of axon chemorepellents class 3 semaphorins (SEMA3s) in skeletal muscle with concomitant upregulation of its receptor Neuropilin 1 (NRP1) in motor axons [243], supporting the idea that diseased muscle is capable of causing retraction of motor axons and NMJ disruption by over-secreting destabilizing factors. Taken together, these findings point to TDP-43 dysregulation in skeletal muscle as a potential mechanism involved in dismantlement of NMJs.

### Glial cells of the central nervous system (CNS)

**Fig. 3:**
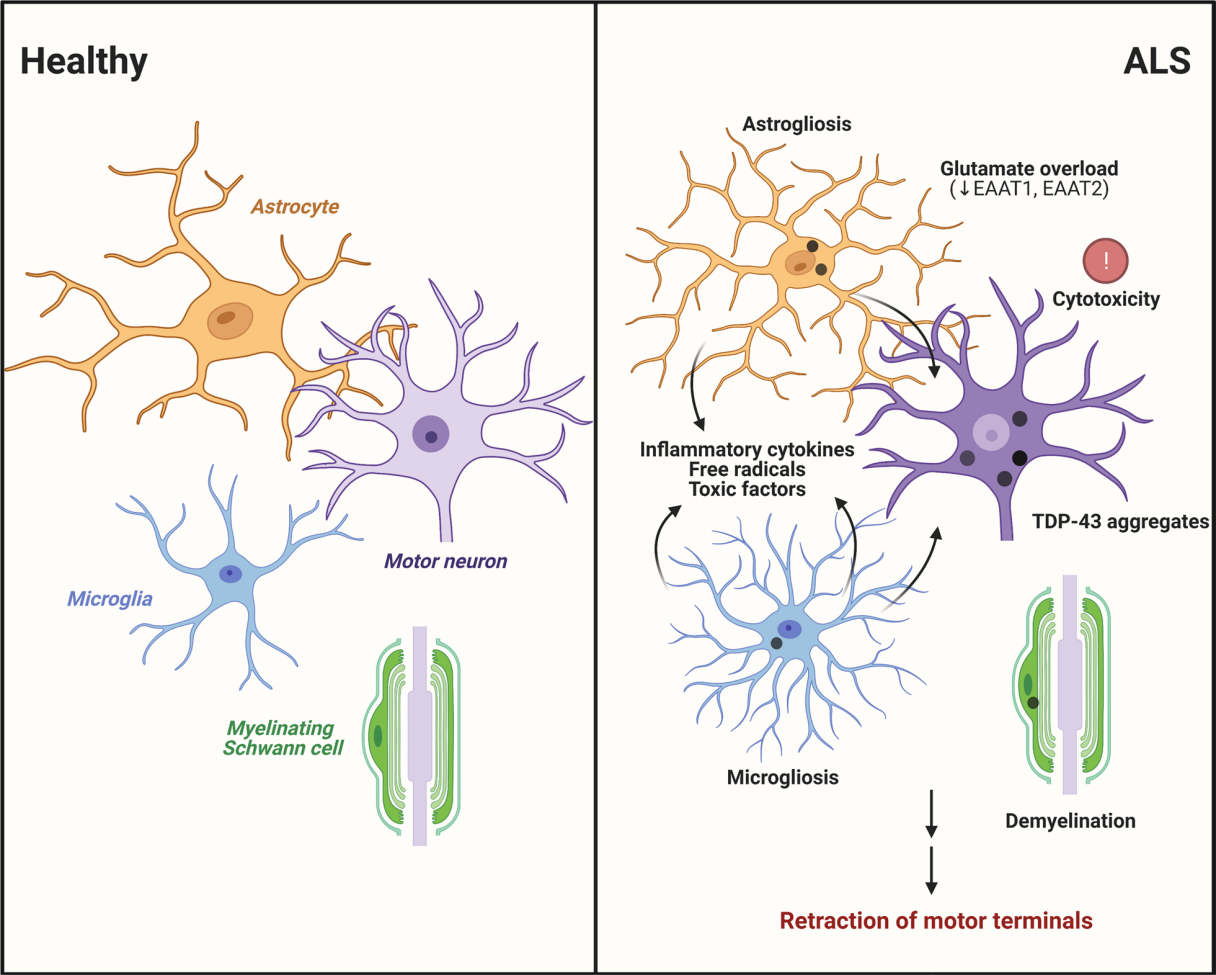
Non-cell autonomous mechanisms exerted by cells interacting with MN cell bodies. TDP-43 aggregates are detected in astrocytes, microglia, oligodendrocytes and myelinating Schwann cells in specimens from individuals with ALS, indicating that TDP-43 also becomes dysregulated in non-neuronal cell types. TDP-43 dysregulation has been associated with demyelination, glial activation (i.e., astrogliosis, microgliosis), and glutamate mishandling by astrocytes, which may trigger functional deficits at the NMJ or cause cytotoxicity leading to the retraction of motor terminals

Non-cell autonomous mechanisms of disease involving glial cells have also become important areas of ALS research. TDP-43 aggregates have been described in astrocytes, microglia and oligodendrocytes of patient specimens [19–21, 244], implying that TDP-43 also becomes dysregulated in non-neuronal cells in ALS. One prominent pathological feature of several neurodegenerative disorders including ALS is the activation of astrocytes and microglia, termed reactive astrogliosis and microgliosis (Fig. 3). This process is thought to create a vulnerable environment for MNs by releasing pro-inflammatory cytokines, free radicals and other neurotoxic factors that exert deleterious effects on MNs [245–247]. Mutations in *TARDBP* were repeatedly shown to increase activation of astrocytes and microglia in mice [56, 248–253]. Additionally, there is evidence that TDP-43-induced glial activation leads to structural defects at the NMJ. In the fruit fly, selective overexpression of wild-type and ALS-associated TDP-43 variants (D169G, G298S, A315T, N345K) in glia triggered NMJ defects, motor deficits and a reduced lifespan [45, 254]. Mechanistically, Lee and colleagues showed that pan-glial overexpression of wild-type TDP-43 caused the upregulation of *Ptp61f*, a gene implicated in inflammation and ER stress signaling pathways [254]. *Ptp61f* knockdown in TDP-43-overexpressing flies suppressed inflammatory cytokines secretion and rescued reduced synaptic button number at NMJs, climbing deficits and lifespan.

In contrast, astrocyte-specific TDP-43 depletion in mice does not cause NMJ denervation or MN loss, but induces an A1-like reactive astrocyte molecular profile, upregulation of C1 complement expression in microglia (a marker of reactive microglia), and reduces the number of mature oligodendrocytes, referred by the authors as “triglial dysfunction” [255]. Similarly, selective depletion of TDP-43 in oligodendrocytes is insufficient to cause NMJ denervation or MN loss, but it is essential for oligodendrocyte survival and myelination [256–258].

Aside from neuroinflammation, impaired glutamate clearing is another proposed mechanism implicating astrocytic dysfunction in ALS. In physiological conditions, astrocytes take up glutamate from excitatory synapses via excitatory amino acid transporter 1 and 2 (EAAT1/2). When uptake is impaired, accumulation of glutamate in the synaptic cleft causes excessive neuronal stimulation, calcium overload and intracellular damage leading to neurodegeneration. This process, termed excitotoxicity, has been proposed to play a role in ALS pathogenesis as per the dying-forward hypothesis. Motor cortex and spinal cord specimens of patients display a significant decrease in EAAT2 [259]. Intriguingly, TDP-43 has been reported to bind to the 3’-UTR of the *EAAT2* transcript in the human brain [96]. Moreover, glial-specific overexpression and depletion of TDP-43/TBPH induces a downregulation of *EAAT1* and *EAAT2* transcript levels in the fruit fly [227]. In transgenic rats, selective overexpression of TDP-43^M337V^ in astrocytes results in a progressive decrease in EAAT1 and EAAT2 immunoreactivity in the spinal cord, and causes denervation atrophy of muscles and MN death [260]. Although ACh is the main neurotransmitter at the NMJ, there is evidence for the involvement of glutamate at this synapse (reviewed in [261]), notably by modulating ACh release via the activation of presynaptic ionotropic receptors [262]. In this regard, one might posit that dysregulation of glutamate handling at spinal excitatory synapses and at the NMJ itself may impact cholinergic transmission or cause cytotoxicity that leads to the retraction of motor terminals.

### Peripheral glial cells

Although the involvement of glia from the CNS has been investigated more extensively, there is also evidence for a contribution of glial cells from the peripheral nervous system in ALS, namely the myelin-forming and terminal Schwann cells (TSCs). Myelin-forming Schwann cells produce the myelin sheath that wraps the axons of MNs to enable rapid saltatory conduction of action potentials. Peripheral nerves of ALS patients often show signs of demyelination [263, 264]. In mice, *TARDBP* knockout in myelin-forming Schwann cells causes severe motor deficits due to impaired formation of paranodal junctions, which maximize nerve conduction velocity in MNs [265]. Two recent studies described the accumulation of abnormally phosphorylated TDP-43 in the cytoplasm of myelin-forming Schwann cells in motor nerve biopsies of ALS patients [263, 266]. It remains unknown if the pathological TDP-43 aggregation in Schwann cells correlates to a decrease in nerve conduction velocity since such clinical studies were not described. However, slow nerve conduction velocity is a clinical hallmark of ALS [267]. Currently, the question remains whether the dysregulation of TDP-43 in myelin-forming Schwann cells can trigger functional deficits at the NMJ leading to its dismantlement.

TSCs, also known as perisynaptic Schwann cells, are considered the third cellular component of the tripartite synapse as they cap motor terminals and provide trophic support to the NMJ [268] (Fig. 2). The TSC is acknowledged as a key player in NMJ maintenance, synaptic transmission and synaptic plasticity [269–271]. TSCs are involved in nerve-muscle reinnervation by guiding the regenerating nerve terminals through extending long processes during synaptic repair [272–274]. For these reasons, their possible contribution to ALS disease mechanisms (where the denervation rate surpasses the reinnervation rate) should not be neglected. TSC dysfunction has been linked to neuromuscular pathology in ALS mice; however, the studies published to date have been performed exclusively in SOD1 mice models [275–277]. Notably, a decrease in TSC number and morphological abnormalities have been described in mutant *SOD1* mice prior to onset of denervation [276]. Other studies have shown impairment of the synaptic decoding abilities of TSCs that could potentially be affecting NMJ repair [275, 277]. TSCs are tuned to their associated presynaptic terminal: they express surface muscarinic receptors that detect ACh upon its release from motor terminals, eliciting a Ca^2+^ response that allows for modulation of synaptic transmission [278]. TSC switching from maintenance to repair mode depends on this ability to detect synaptic transmission, which was shown to be dysregulated in SOD1^G37R^ mice [275, 277]. Consequently, TSCs were unable to adopt a phagocytic phenotype and extended abnormal processes at denervated NMJs in symptomatic animals [277]. Since reinnervation mechanisms appear to be deficient in ALS models, including several TDP-43 models (Table 1), the question arises as to whether such deficits could be linked to the failure of TSCs to properly decode the innervation state of the NMJ. Future studies in non-SOD1 genetic models are needed to determine the broader implication of this mechanism in ALS. In particular, the role of TDP-43 in TSCs is still largely unknown and, to our knowledge, TSC phenotypes have not yet been investigated in TDP-43 models.

## Dying-back, dying-forward or both?

ALS is a complex disease, most likely caused by a combination of genetic and environmental factors as well as age-related changes. As summarized in this review, disturbances of several cellular pathways have been suggested to play a role, making it difficult to reconcile the many fundamental features of ALS into one disease model. This challenge is further complicated by the heterogeneity of the clinical manifestations of ALS (including its close association with FTD) and the presence of multisystem impairments.

In the sections above, we provided an overview of the potential mechanisms underlying NMJ disruption in ALS mediated by dysregulated TDP-43 in different cell types. Some of these studies argued that defects may originate distally, such as the evidence for a toxic role of skeletal muscle [220–222], the involvement of programmed axon death [66, 171, 172], and alterations in factors implicated in NMJ formation and maintenance (e.g., agrin, MAP1B, AChE) [102, 103, 110]. In contrast, findings of dysregulated expression of synaptic genes [90, 98] or glutamate transporters by astrocytes [259, 260] are consistent with the excitotoxic mechanisms proposed by the dying-forward hypothesis. Although several studies summarized here established a mechanistic link between TDP-43 and NMJ defects, few examined the chronological relationship between these events and, as such, they do not directly support one or the other hypothesis. Further, some proposed mechanisms (e.g., impaired RNA processing, DNA damage, mitochondrial dysfunction) could occur in both upper and lower MNs, with potentially broad pathological consequences beyond NMJ disruption. More detailed investigations are needed to determine the spatiotemporal progression of pathology in this disease. For instance, in vivo studies comparing the timing of brain, spinal cord, nerve and NMJ pathology and the onset of symptoms would provide further insights into the series of events leading to the manifestation of an ALS-like phenotype.

At present, beyond the old dichotomy of “dying-back” versus “dying-forward”, we can speculate that several pathologies may occur simultaneously early in the disease course. For instance, excitotoxic effects exerted by upper MNs may be potentiated by lower MNs’ inability to cope with additional stressors (e.g., due to deficient DNA repair) and accelerate the dismantling of already-vulnerable NMJs (e.g., due to dysfunctional TSCs). An additional consideration is how the primary site of onset is defined, which can lead to different interpretations with regards to the direction of disease progression. For instance, let us consider the suggestion that dysregulation of key pathways in MN cell bodies triggers the dismantling of distal structures. In this scenario, the primary dysfunction originates from cell bodies and propagates anterogradely to affect the NMJs (dying-forward), but denervation and axonal fragmentation may occur before the MN cell bodies degenerate and symptoms become apparent (dying-back). This example highlights the importance of considering the multiple scales at which pathogenic changes can take place (spanning from molecular to macroscopic scales). Finally, we can hypothesize that different genetic or clinical subtypes of ALS patients may present distinct predominant dying-back or dying-forward patterns of neurodegeneration, with pathology being more pronounced in the motor cortex or in distal structures at early stages of disease. This integrated view could partially explain the heterogeneity of clinical presentations of ALS patients, including the variability in the site of symptom onset (i.e., spinal vs. bulbar ALS) and the presence of extra-motor manifestations [279].

## Conclusion

ALS is a complex and heterogeneous disease, most likely caused by a combination of factors. It is becoming clear that pathological dysregulation of TDP-43, not only in MNs but also in non-neuronal cells such as skeletal muscle and glial cells, plays an important role in disease pathogenesis. Abnormalities in RNA processing, DNA repair, mitochondrial function, axonal transport and protein aggregation are TDP-43-mediated changes that may be important contributors to NMJ disruption and MN loss. The detection of aggregates in several types of cells showing pathogenic changes in ALS (MNs, astrocytes, microglia, oligodendrocytes, myelinating Schwann cells, and skeletal muscle) may indicate that the impact of TDP-43 dysregulation in ALS is underestimated. While TDP-43 is expressed ubiquitously, little is known about the consequences ALS-linked TDP-43 variants in non-neuronal cells, particularly those forming the NMJ. A better understanding of when and how NMJ defects arise in ALS will be critical for the development of therapies that can meaningfully delay or halt functional decline. In particular, therapeutics targeting NMJs would avoid the need for molecules that cross the blood-brain barrier or having patients undergo invasive drug delivery procedures. Combination therapies targeting several pathways, rather than individual targets, may also be a promising avenue given the multifactorial nature of this disease.

## Data Availability

Not applicable.

## Abbreviations

ACh: Acetylcholine
AChE: Acetylcholine esterase
*AGRN*: Agrin
ALS: Amyotrophic lateral sclerosis
axon-seq: Axon sequencing
BMP: Bone morphogenetic protein
CNS: Central nervous system
*C9ORF72*: Chromosome 9 open reading frame 72
SEMA3s: Class 3 semaphorins
Dlg: Discs-large
DSBs: Double-stranded DNA breaks
DDR: DNA damage response
EAAT1/2: Excitatory amino acid transporter 1 and 2
FMRP: Fragile X mental retardation protein
FTD: Frontotemporal dementia
*FUS*: Fused in sarcomas
HDAC4: Histone deacetylase 4
IBM: Inclusion bodies myositis
iPSCs: Induced pluripotent stem cells
IGF-1: Insulin-like growth factor 1
MN: Motor neuron
mESC: Mouse embryonic stem cell
*Nlgn1*: Neuroligin 1
NMJ: Neuromuscular junction
NRP1: Neuropilin 1
NEHJ: Non-homologous end joining
ROS: Reactive oxygen species
RNPs: Ribonucleoproteins
RPs: Ribosomal proteins
SNPs: Single nucleotide polymorphisms
*STMN2*: Stathmin-2
*SARM1*: Sterile Alpha and TIR Motif-Containing 1
*SOD1*: Superoxide dismutase 1
*VAMP1*: Synaptobrevin 1
*SNAP25*: Synaptosomal-associated protein 25
TSCs: Terminal Schwann cells
*TARDBP*: Transactive-response DNA binding protein

## References

[1] Charcot J-M, Joffroy A (1869). Deux cas d’atrophie musculaire progressive avec lésions de la substance grise et des faiseaux antéro-latéraux de la moelle épinière. Arch Physiol Norm Pathol..

[2] Neudert C, Oliver D, Wasner M, Borasio GD (2001). The course of the terminal phase in patients with amyotrophic lateral sclerosis. J Neurol.

[3] del Aguila MA, Longstreth WT, McGuire V, Koepsell TD, van Belle G (2003). Prognosis in amyotrophic lateral sclerosis: a population-based study. Neurology.

[4] Bensimon G, Lacomblez L, Meininger V (1994). A controlled trial of riluzole in amyotrophic lateral sclerosis. N Engl J Med.

[5] Lacomblez L, Bensimon G, Meininger V, Leigh P, Guillet P (1996). Dose-ranging study of riluzole in amyotrophic lateral sclerosis. Lancet.

[6] Hinchcliffe M, Smith A (2017). Riluzole: real-world evidence supports significant extension of median survival times in patients with amyotrophic lateral sclerosis. Degener Neurol Neuromuscul Dis.

[7] Abe K, Aoki M, Tsuji S, Itoyama Y, Sobue G, Togo M (2017). Safety and efficacy of edaravone in well defined patients with amyotrophic lateral sclerosis: a randomised, double-blind, placebo-controlled trial. Lancet Neurol.

[8] Sakata T, Palumbo J, Akimoto M, Tanaka M (2016). A long-term safety and efficacy extension study of patients diagnosed with amyotrophic lateral sclerosis (ALS) and treated with edaravone (MCI-186). Neurology.

[9] Paganoni S, Macklin EA, Hendrix S, Berry JD, Elliott MA, Maiser S (2020). Trial of sodium phenylbutyrate–taurursodiol for amyotrophic lateral sclerosis. N Engl J Med.

[10] Paganoni S, Knowlton N, Hendrix K, Ellison N, Dickson S, Hendrix S (2020). Long-term treatment with AMX0035 in the open-label extension of CENTAUR, a randomized controlled trial in individuals with amyotrophic lateral sclerosis. Muscle Nerve.

[11] Paganoni S, Hendrix S, Dickson SP, Knowlton N, Macklin EA, Berry JD (2021). Long-term survival of participants in the CENTAUR trial of sodium phenylbutyrate-taurursodiol in amyotrophic lateral sclerosis. Muscle Nerve.

[12] Al-Chalabi ABRH (2017). Amyotrophic lateral sclerosis. N Engl J Med.

[13] Chen S, Sayana P, Zhang X, Le W (2013). Genetics of amyotrophic lateral sclerosis: an update. Mol Neurodegener.

[14] Gitcho MA, Baloh RH, Chakraverty S, Mayo K, Norton JB, Levitch D (2008). TDP-43 A315T mutation in familial motor neuron disease. Ann Neurol.

[15] Kabashi E, Valdmanis PN, Dion P, Spiegelman D, McConkey BJ, Velde C, Vande (2008). TARDBP mutations in individuals with sporadic and familial amyotrophic lateral sclerosis. Nat Genet.

[16] Sreedharan J, Blair IP, Tripathi VB, Hu X, Vance C, Rogelj B (2008). TDP-43 mutations in familial and sporadic amyotrophic lateral sclerosis. Science.

[17] Van Deerlin VM, Leverenz JB, Bekris LM, Bird TD, Yuan W, Elman LB (2008). TARDBP mutations in amyotrophic lateral sclerosis with TDP-43 neuropathology: a genetic and histopathological analysis. Lancet Neurol.

[18] Lagier-Tourenne C, Cleveland DW, Rethinking ALS (2009). The FUS about TDP-43. Cell.

[19] Arai T, Hasegawa M, Akiyama H, Ikeda K, Nonaka T, Mori H (2006). TDP-43 is a component of ubiquitin-positive tau-negative inclusions in frontotemporal lobar degeneration and amyotrophic lateral sclerosis. Biochem Biophys Res Commun.

[20] Neumann M, Sampathu DM, Kwong LK, Truax AC, Micsenyi MC, Chou TT (2006). Ubiquitinated TDP-43 in frontotemporal lobar degeneration and amyotrophic lateral sclerosis. Science.

[21] Mackenzie IRA, Bigio EH, Ince PG, Geser F, Neumann M, Cairns NJ (2007). Pathological TDP-43 distinguishes sporadic amyotrophic lateral sclerosis from amyotrophic lateral sclerosis with SOD1 mutations. Ann Neurol.

[22] Felice KJ (1997). A longitudinal study comparing thenar motor unit number estimates to other quantitative tests in patients with amyotrophic lateral sclerosis. Muscle Nerve.

[23] Fischer LR, Culver DG, Tennant P, Davis AA, Wang M, Castellano-Sanchez A (2004). Amyotrophic lateral sclerosis is a distal axonopathy: evidence in mice and man. Exp Neurol.

[24] Killian JM, Wilfong AA, Burnett L, Appel SH, Boland D (1994). Decremental motor responses to repetitive nerve stimulation in ALS. Muscle Nerve.

[25] Frey D, Schneider C, Xu L, Borg J, Spooren W, Caroni P (2000). Early and selective loss of neuromuscular synapse subtypes with low sprouting competence in motoneuron diseases. J Neurosci.

[26] Maselli RA, Wollman RL, Leung C, Distad B, Palombi S, Richman DP (1993). Neuromuscular transmission in amyotrophic lateral sclerosis. Muscle Nerve.

[27] Vucic S (2006). Novel threshold tracking techniques suggest that cortical hyperexcitability is an early feature of motor neuron disease. Brain.

[28] Vucic S, Nicholson GA, Kiernan MC (2008). Cortical hyperexcitability may precede the onset of familial amyotrophic lateral sclerosis. Brain.

[29] Geevasinga N, Menon P, Nicholson GA, Ng K, Howells J, Kril JJ (2015). Cortical function in asymptomatic carriers and patients with C9orf72 amyotrophic lateral sclerosis. JAMA Neurol.

[30] Menon P, Kiernan MC, Vucic S (2015). Cortical hyperexcitability precedes lower motor neuron dysfunction in ALS. Clin Neurophysiol.

[31] Blizzard CA, Southam KA, Dawkins E, Lewis KE, King AE, Clark JA (2015). Identifying the primary site of pathogenesis in amyotrophic lateral sclerosis: vulnerability of lower motor neurons to proximal excitotoxicity. Dis Model Mech.

[32] Dadon-Nachum M, Melamed E, Offen D (2011). The “dying-back” phenomenon of motor neurons in ALS. J Mol Neurosci.

[33] Moloney EB, de Winter F, Verhaagen J (2014). ALS as a distal axonopathy: molecular mechanisms affecting neuromuscular junction stability in the presymptomatic stages of the disease. Front Neurosci.

[34] Geevasinga N, Menon P, Özdinler PH, Kiernan MC, Vucic S (2016). Pathophysiological and diagnostic implications of cortical dysfunction in ALS. Nat Rev Neurol.

[35] Eisen A, Braak H, Del Tredici K, Lemon R, Ludolph AC, Kiernan MC (2017). Cortical influences drive amyotrophic lateral sclerosis. J Neurol Neurosurg Psychiatry.

[36] Geser F, Fellner L, Haybaeck J, Wenning GK (2020). Development of neurodegeneration in amyotrophic lateral sclerosis: from up or down?. J Neural Transm.

[37] Feiguin F, Godena VK, Romano G, D’Ambrogio A, Klima R, Baralle FE (2009). Depletion of TDP-43 affects *Drosophila motoneurons* terminal synapsis and locomotive behavior. FEBS Lett.

[38] Li Y, Ray P, Rao EJ, Shi C, Guo W, Chen X (2010). A *Drosophila* model for TDP-43 proteinopathy. Proc Natl Acad Sci U S A.

[39] Godena VK, Romano G, Romano M, Appocher C, Klima R, Buratti E (2011). TDP-43 regulates *Drosophila* neuromuscular junctions growth by modulating futsch/MAP1B levels and synaptic microtubules organization. PLoS ONE.

[40] Wang J-W, Brent JR, Tomlinson A, Shneider NA, McCabe BD (2011). The ALS-associated proteins FUS and TDP-43 function together to affect *Drosophila* locomotion and life span. J Clin Invest.

[41] Donde A, Sun M, Ling JP, Braunstein KE, Pang B, Wen X (2019). Splicing repression is a major function of TDP-43 in motor neurons. Acta Neuropathol.

[42] Estes PS, Boehringer A, Zwick R, Tang JE, Grigsby B, Zarnescu DC (2011). Wild-type and A315T mutant TDP-43 exert differential neurotoxicity in a Drosophila model of ALS. Hum Mol Genet.

[43] Lin M-J, Cheng C-W, Shen C-KJ (2011). Neuronal function and dysfunction of *Drosophila* dTDP. PLoS ONE.

[44] Diaper DC, Adachi Y, Sutcliffe B, Humphrey DM, Elliott CJH, Stepto A (2013). Loss and gain of *Drosophila* TDP-43 impair synaptic efficacy and motor control leading to age-related neurodegeneration by loss-of-function phenotypes. Hum Mol Genet.

[45] Estes PS, Daniel SG, Mccallum AP, Boehringer AV, Sukhina AS, Zwick RA (2013). Motor neurons and glia exhibit specific individualized responses to TDP-43 expression in a *Drosophila* model of amyotrophic lateral sclerosis. DMM Dis Model Mech.

[46] Romano G, Klima R, Buratti E, Verstreken P, Baralle FE, Feiguin F (2014). Chronological requirements of TDP-43 function in synaptic organization and locomotive control. Neurobiol Dis.

[47] Deshpande M, Feiger Z, Shilton AK, Luo CC, Silverman E, Rodal AA (2016). Role of BMP receptor traffic in synaptic growth defects in an ALS model. Mol Biol Cell.

[48] Kabashi E, Lin L, Tradewell ML, Dion PA, Bercier V, Bourgouin P (2010). Gain and loss of function of ALS-related mutations of TARDBP (TDP-43) cause motor deficits in vivo. Hum Mol Genet.

[49] Kabashi E, Bercier V, Lissouba A, Liao M, Brustein E, Rouleau GA (2011). FUS and TARDBP but not SOD1 interact in genetic models of amyotrophic lateral sclerosis. PLoS Genet.

[50] Dzieciolowska S, Drapeau P, Armstrong GAB (2017). Augmented quantal release of acetylcholine at the vertebrate neuromuscular junction following tdp-43 depletion. PLoS ONE.

[51] Bose P, Armstrong GAB, Drapeau P (2019). Neuromuscular junction abnormalities in a zebrafish loss-of-function model of TDP-43. J Neurophysiol.

[52] Campanari M-L, Marian A, Ciura S, Kabashi E (2021). TDP-43 regulation of AChE expression can mediate ALS-like phenotype in zebrafish. Cells.

[53] Armstrong GAB, Drapeau P (2013). Calcium channel agonists protect against neuromuscular dysfunction in a genetic model of TDP-43 mutation in ALS. J Neurosci.

[54] Patten SA, Aggad D, Martinez J, Tremblay E, Petrillo J, Armstrong GAB (2017). Neuroleptics as therapeutic compounds stabilizing neuromuscular transmission in amyotrophic lateral sclerosis. JCI Insight.

[55] Wegorzewska I, Bell S, Cairns NJ, Miller TM, Baloh RH (2009). TDP-43 mutant transgenic mice develop features of ALS and frontotemporal lobar degeneration. Proc Natl Acad Sci U S A.

[56] Zhou H, Huang C, Chen H, Wang D, Landel CP, Xia PY (2010). Transgenic rat model of neurodegeneration caused by mutation in the TDP gene. PLoS Genet.

[57] Huang S-L, Wu L-S, Lee M, Chang C-W, Cheng W-C, Fang Y-S (2020). A robust TDP-43 knock-in mouse model of ALS. Acta Neuropathol Commun.

[58] Sleigh JN, Tosolini AP, Gordon D, Devoy A, Fratta P, Fisher EMC (2020). Mice carrying ALS mutant TDP-43, but not mutant FUS, display in vivo defects in axonal transport of signaling endosomes. Cell Rep.

[59] Swarup V, Phaneuf D, Bareil C, Robertson J, Rouleau GA, Kriz J (2011). Pathological hallmarks of amyotrophic lateral sclerosis/frontotemporal lobar degeneration in transgenic mice produced with TDP-43 genomic fragments. Brain.

[60] Arnold ES, Ling S-C, Huelga SC, Lagier-Tourenne C, Polymenidou M, Ditsworth D (2013). ALS-linked TDP-43 mutations produce aberrant RNA splicing and adult-onset motor neuron disease without aggregation or loss of nuclear TDP-43. Proc Natl Acad Sci U S A.

[61] Mitchell JC, Constable R, So E, Vance C, Scotter E, Glover L (2015). Wild type human TDP-43 potentiates ALS-linked mutant TDP-43 driven progressive motor and cortical neuron degeneration with pathological features of ALS. Acta Neuropathol Commun.

[62] Chand KK, Lee KM, Lee JD, Qiu H, Willis EF, Lavidis NA (2018). Defects in synaptic transmission at the neuromuscular junction precede motor deficits in a TDP-43 Q331K transgenic mouse model of amyotrophic lateral sclerosis. FASEB J.

[63] Ebstein SY, Yagudayeva I, Shneider NA (2019). Mutant TDP-43 causes early-stage dose-dependent motor neuron degeneration in a TARDBP knockin mouse model of ALS. Cell Rep.

[64] Gordon D, Dafinca R, Scaber J, Alegre-Abarrategui J, Farrimond L, Scott C (2019). Single-copy expression of an amyotrophic lateral sclerosis-linked TDP-43 mutation (M337V) in BAC transgenic mice leads to altered stress granule dynamics and progressive motor dysfunction. Neurobiol Dis.

[65] Williamson MG, Finelli MJ, Sleigh JN, Reddington A, Gordon D, Talbot K (2019). Neuronal over-expression of Oxr1 is protective against ALS-associated mutant TDP-43 mislocalisation in motor neurons and neuromuscular defects in vivo. Hum Mol Genet.

[66] White MA, Lin Z, Kim E, Henstridge CM, Pena Altamira E, Hunt CK (2019). Sarm1 deletion suppresses TDP-43-linked motor neuron degeneration and cortical spine loss. Acta Neuropathol Commun.

[67] Osaki T, Uzel SGM, Kamm RD (2018). Microphysiological 3D model of amyotrophic lateral sclerosis (ALS) from human iPS-derived muscle cells and optogenetic motor neurons. Sci Adv.

[68] Pereira JD, DuBreuil DM, Devlin A-C, Held A, Sapir Y, Berezovski E (2021). Human sensorimotor organoids derived from healthy and amyotrophic lateral sclerosis stem cells form neuromuscular junctions. Nat Commun.

[69] Ou SH, Wu F, Harrich D, García-Martínez LF, Gaynor RB (1995). Cloning and characterization of a novel cellular protein, TDP-43, that binds to human immunodeficiency virus type 1 TAR DNA sequence motifs. J Virol.

[70] Buratti E (2001). Nuclear factor TDP-43 and SR proteins promote in vitro and in vivo CFTR exon 9 skipping. EMBO J.

[71] Mitra J, Guerrero EN, Hegde PM, Liachko NF, Wang H, Vasquez V (2019). Motor neuron disease-associated loss of nuclear TDP-43 is linked to DNA double-strand break repair defects. Proc Natl Acad Sci U S A.

[72] Konopka A, Whelan DR, Jamali MS, Perri E, Shahheydari H, Toth RP (2020). Impaired NHEJ repair in amyotrophic lateral sclerosis is associated with TDP-43 mutations. Mol Neurodegener.

[73] Chu J-F, Majumder P, Chatterjee B, Huang S-L, Shen C-KJ (2019). TDP-43 regulates coupled dendritic mRNA transport-translation processes in co-operation with FMRP and Staufen1. Cell Rep.

[74] Alami NH, Smith RB, Carrasco MA, Williams LA, Winborn CS, Han SSW (2014). Axonal transport of TDP-43 mRNA granules is impaired by ALS-causing mutations. Neuron.

[75] Fallini C, Bassell GJ, Rossoll W (2012). The ALS disease protein TDP-43 is actively transported in motor neuron axons and regulates axon outgrowth. Hum Mol Genet.

[76] Gopal PP, Nirschl JJ, Klinman E, Holzbaur ELF (2017). Amyotrophic lateral sclerosis-linked mutations increase the viscosity of liquid-like TDP-43 RNP granules in neurons. Proc Natl Acad Sci U S A.

[77] Freibaum BD, Chitta RK, High AA, Taylor JP (2010). Global analysis of TDP-43 interacting proteins reveals strong association with RNA splicing and translation machinery. J Proteome Res.

[78] Wang I-F, Wu L-S, Chang H-Y, Shen C-KJ (2008). TDP-43, the signature protein of FTLD-U, is a neuronal activity-responsive factor. J Neurochem.

[79] Buratti E, De Conti L, Stuani C, Romano M, Baralle M, Baralle F (2010). Nuclear factor TDP-43 can affect selected microRNA levels. FEBS J.

[80] Kawahara Y, Mieda-Sato A (2012). TDP-43 promotes microRNA biogenesis as a component of the Drosha and Dicer complexes. Proc Natl Acad Sci U S A.

[81] Colombrita C, Zennaro E, Fallini C, Weber M, Sommacal A, Buratti E (2009). TDP-43 is recruited to stress granules in conditions of oxidative insult. J Neurochem.

[82] Liu-Yesucevitz L, Bilgutay A, Zhang Y-J, Vanderwyde T, Citro A, Mehta T (2010). Tar DNA binding protein-43 (TDP-43) associates with stress granules: analysis of cultured cells and pathological brain tissue. PLoS ONE.

[83] Dewey CM, Cenik B, Sephton CF, Dries DR, Mayer P, Good SK (2011). TDP-43 is directed to stress granules by sorbitol, a novel physiological osmotic and oxidative stressor. Mol Cell Biol.

[84] McDonald KK, Aulas A, Destroismaisons L, Pickles S, Beleac E, Camu W (2011). TAR DNA-binding protein 43 (TDP-43) regulates stress granule dynamics via differential regulation of G3BP and TIA-1. Hum Mol Genet.

[85] Asakawa K, Handa H, Kawakami K (2020). Optogenetic modulation of TDP-43 oligomerization accelerates ALS-related pathologies in the spinal motor neurons. Nat Commun.

[86] Mitsuzawa S, Suzuki N, Akiyama T, Ishikawa M, Sone T, Kawada J (2021). Reduced PHOX2B stability causes axonal growth impairment in motor neurons with TARDBP mutations. Stem Cell Reports.

[87] White MA, Kim E, Duffy A, Adalbert R, Phillips BU, Peters OM (2018). TDP-43 gains function due to perturbed autoregulation in a Tardbp knock-in mouse model of ALS-FTD. Nat Neurosci.

[88] Fratta P, Sivakumar P, Humphrey J, Lo K, Ricketts T, Oliveira H (2018). Mice with endogenous TDP-43 mutations exhibit gain of splicing function and characteristics of amyotrophic lateral sclerosis. EMBO J.

[89] Watanabe S, Oiwa K, Murata Y, Komine O, Sobue A, Endo F (2020). ALS-linked TDP-43M337V knock-in mice exhibit splicing deregulation without neurodegeneration. Mol Brain.

[90] Polymenidou M, Lagier-Tourenne C, Hutt KR, Huelga SC, Moran J, Liang TY (2011). Long pre-mRNA depletion and RNA missplicing contribute to neuronal vulnerability from loss of TDP-43. Nat Neurosci.

[91] Ling JP, Pletnikova O, Troncoso JC, Wong PC (2015). TDP-43 repression of nonconserved cryptic exons is compromised in ALS-FTD. Science.

[92] Tan Q, Yalamanchili HK, Park J, De Maio A, Lu HC, Wan YW (2016). Extensive cryptic splicing upon loss of RBM17 and TDP43 in neurodegeneration models. Hum Mol Genet.

[93] Humphrey J, Emmett W, Fratta P, Isaacs AM, Plagnol V (2017). Quantitative analysis of cryptic splicing associated with TDP-43 depletion. BMC Med Genom.

[94] Brown A, Wilkins OG, Keuss MJ, Hill SE, Zanovello M, Lee WC (2022). TDP-43 loss and ALS-risk SNPs drive mis-splicing and depletion of UNC13A. Nature.

[95] Sephton CF, Cenik C, Kucukural A, Dammer EB, Cenik B, Han Y (2011). Identification of neuronal RNA targets of TDP-43-containing ribonucleoprotein complexes. J Biol Chem.

[96] Tollervey JR, Curk T, Rogelj B, Briese M, Cereda M, Kayikci M (2011). Characterizing the RNA targets and position-dependent splicing regulation by TDP-43. Nat Neurosci.

[97] Xiao S, Sanelli T, Dib S, Sheps D, Findlater J, Bilbao J (2011). RNA targets of TDP-43 identified by UV-CLIP are deregulated in ALS. Mol Cell Neurosci.

[98] Mishra M, Paunesku T, Woloschak GE, Siddique T, Zhu L, Lin S (2007). Gene expression analysis of frontotemporal lobar degeneration of the motor neuron disease type with ubiquitinated inclusions. Acta Neuropathol.

[99] Ma XR, Prudencio M, Koike Y, Vatsavayai SC, Kim G, Harbinski F (2022). TDP-43 represses cryptic exon inclusion in the FTD–ALS gene UNC13A. Nature.

[100] Honda D, Ishigaki S, Iguchi Y, Fujioka Y, Udagawa T, Masuda A (2014). The ALS/FTLD-related RNA-binding proteins TDP-43 and FUS have common downstream RNA targets in cortical neurons. FEBS Open Bio.

[101] Gautam M, Noakes PG, Moscoso L, Rupp F, Scheller RH, Merlie JP (1996). Defective neuromuscular synaptogenesis in agrin-deficient mutant mice. Cell.

[102] Collins MA, An J, Hood BL, Conrads TP, Bowser RP (2015). Label-free LC–MS/MS proteomic analysis of cerebrospinal fluid identifies protein/pathway alterations and candidate biomarkers for amyotrophic lateral sclerosis. J Proteome Res.

[103] Coyne AN, Siddegowda BB, Estes PS, Johannesmeyer J, Kovalik T, Daniel SG (2014). FUTSCH/MAP1B mRNA is a translational target of TDP-43 and is neuroprotective in a *Drosophila* model of amyotrophic lateral sclerosis. J Neurosci.

[104] Strah N, Romano G, Introna C, Klima R, Marzullo M, Ciapponi L (2020). TDP-43 promotes the formation of neuromuscular synapses through the regulation of disc-large expression in *Drosophila* skeletal muscles. BMC Biol.

[105] Roos J, Hummel T, Ng N, Klämbt C, Davis GW (2000). *Drosophila* Futsch regulates synaptic microtubule organization and is necessary for synaptic growth. Neuron.

[106] Dudel J, Heckmann M (1999). Desensitization reduces amplitudes of quantal end-plate currents after a single preceding end-plate current in mouse muscle. Pflug Arch Eur J Physiol.

[107] Adler M, Manley HA, Purcell AL, Deshpande SS, Hamilton TA, Kan RK (2004). Reduced acetylcholine receptor density, morphological remodeling, and butyrylcholinesterase activity can sustain muscle function in acetylcholinesterase knockout mice. Muscle Nerve.

[108] Lin W, Dominguez B, Yang J, Aryal P, Brandon EP, Gage FH (2005). Neurotransmitter acetylcholine negatively regulates neuromuscular synapse formation by a Cdk5-dependent mechanism. Neuron.

[109] Girard E, Bernard V, Camp S, Taylor P, Krejci E, Molgó J (2006). Remodeling of the neuromuscular junction in mice with deleted exons 5 and 6 of acetylcholinesterase. J Mol Neurosci.

[110] Maniatis S, Äijö T, Vickovic S, Braine C, Kang K, Mollbrink A (2019). Spatiotemporal dynamics of molecular pathology in amyotrophic lateral sclerosis. Science.

[111] Kawaguchi T, Rollins MG, Moinpour M, Morera AA, Ebmeier CC, Old WM (2020). Changes to the TDP-43 and FUS interactomes induced by DNA damage. J Proteome Res.

[112] Hill SJ, Mordes DA, Cameron LA, Neuberg DS, Landini S, Eggan K (2016). Two familial ALS proteins function in prevention/repair of transcription-associated DNA damage. Proc Natl Acad Sci U S A.

[113] Giannini M, Bayona-Feliu A, Sproviero D, Barroso SI, Cereda C, Aguilera A (2020). TDP-43 mutations link amyotrophic lateral sclerosis with R-loop homeostasis and R loop-mediated DNA damage. PLOS Genet.

[114] Guerrero EN, Mitra J, Wang H, Rangaswamy S, Hegde PM, Basu P (2019). Amyotrophic lateral sclerosis-associated TDP-43 mutation Q331K prevents nuclear translocation of XRCC4-DNA ligase 4 complex and is linked to genome damage-mediated neuronal apoptosis. Hum Mol Genet.

[115] Wu C, Jin L, Wang I, Wei W, Ho P, Liu Y (2020). HDAC1 dysregulation induces aberrant cell cycle and DNA damage in progress of TDP-43 proteinopathies. EMBO Mol Med.

[116] Stein D, Toiber D (2017). DNA damage and neurodegeneration: the unusual suspect. Neural Regen Res.

[117] Walker AK, Spiller KJ, Ge G, Zheng A, Xu Y, Zhou M (2015). Functional recovery in new mouse models of ALS/FTLD after clearance of pathological cytoplasmic TDP-43. Acta Neuropathol.

[118] Spiller KJ, Cheung CJ, Restrepo CR, Kwong LK, Stieber AM, Trojanowski JQ (2016). Selective motor neuron resistance and recovery in a new inducible mouse model of TDP-43 proteinopathy. J Neurosci.

[119] Naumann M, Pal A, Goswami A, Lojewski X, Japtok J, Vehlow A (2018). Impaired DNA damage response signaling by FUS-NLS mutations leads to neurodegeneration and FUS aggregate formation. Nat Commun.

[120] Turrens JF (2003). Mitochondrial formation of reactive oxygen species. J Physiol.

[121] Sasaki S, Iwata M (1999). Ultrastructural change of synapses of Betz cells in patients with amyotrophic lateral sclerosis. Neurosci Lett.

[122] Ferrante RJ, Browne SE, Shinobu LA, Bowling AC, Baik MJ, MacGarvey U (1997). Evidence of increased oxidative damage in both sporadic and familial amyotrophic lateral sclerosis. J Neurochem.

[123] Abe K, Pan L-H, Watanabe M, Kato T, Itoyama Y (1995). Induction of nitrotyrosine-like immunoreactivity in the lower motor neuron of amyotrophic lateral sclerosis. Neurosci Lett.

[124] Duan W, Li X, Shi J, Guo Y, Li Z, Li C (2010). Mutant TAR DNA-binding protein-43 induces oxidative injury in motor neuron-like cell. Neuroscience.

[125] Hong K, Li Y, Duan W, Guo Y, Jiang H, Li W (2012). Full-length TDP-43 and its C-terminal fragments activate mitophagy in NSC34 cell line. Neurosci Lett.

[126] Onesto E, Colombrita C, Gumina V, Borghi MO, Dusi S, Doretti A (2016). Gene-specific mitochondria dysfunctions in human TARDBP and C9ORF72 fibroblasts. Acta Neuropathol Commun.

[127] Zanini G, Selleri V, Nasi M, De Gaetano A, Martinelli I, Gianferrari G (2022). Mitochondrial and endoplasmic reticulum alterations in a case of amyotrophic lateral sclerosis caused by TDP-43 A382T mutation. Int J Mol Sci.

[128] Lu J, Duan W, Guo Y, Jiang H, Li Z, Huang J (2012). Mitochondrial dysfunction in human TDP-43 transfected NSC34 cell lines and the protective effect of dimethoxy curcumin. Brain Res Bull.

[129] Dafinca R, Barbagallo P, Farrimond L, Candalija A, Scaber J, Ababneh NA (2020). Impairment of mitochondrial calcium buffering links mutations in C9ORF72 and TARDBP in iPS-derived motor neurons from patients with ALS/FTD. Stem Cell Reports.

[130] Mori F, Tanji K, Zhang H-X, Nishihira Y, Tan C-F, Takahashi H (2008). Maturation process of TDP-43-positive neuronal cytoplasmic inclusions in amyotrophic lateral sclerosis with and without dementia. Acta Neuropathol.

[131] Wang W, Li L, Lin W-L, Dickson DW, Petrucelli L, Zhang T (2013). The ALS disease-associated mutant TDP-43 impairs mitochondrial dynamics and function in motor neurons. Hum Mol Genet.

[132] Wang W, Wang L, Lu J, Siedlak SL, Fujioka H, Liang J (2016). The inhibition of TDP-43 mitochondrial localization blocks its neuronal toxicity. Nat Med.

[133] Shan X, Chiang P-M, Price DL, Wong PC (2010). Altered distributions of Gemini of coiled bodies and mitochondria in motor neurons of TDP-43 transgenic mice. Proc Natl Acad Sci U S A.

[134] Xu Y-F, Gendron TF, Zhang Y-J, Lin W-L, D’Alton S, Sheng H (2010). Wild-type human TDP-43 expression causes TDP-43 phosphorylation, mitochondrial aggregation, motor deficits, and early mortality in transgenic mice. J Neurosci.

[135] Zuo X, Zhou J, Li Y, Wu K, Chen Z, Luo Z (2021). TDP-43 aggregation induced by oxidative stress causes global mitochondrial imbalance in ALS. Nat Struct Mol Biol.

[136] Altman T, Ionescu A, Ibraheem A, Priesmann D, Gradus-Pery T, Farberov L (2021). Axonal TDP-43 condensates drive neuromuscular junction disruption through inhibition of local synthesis of nuclear encoded mitochondrial proteins. Nat Commun.

[137] Stribl C, Samara A, Trümbach D, Peis R, Neumann M, Fuchs H (2014). Mitochondrial dysfunction and decrease in body weight of a transgenic knock-in mouse model for TDP-43. J Biol Chem.

[138] Magrané J, Cortez C, Gan W-B, Manfredi G (2014). Abnormal mitochondrial transport and morphology are common pathological denominators in SOD1 and TDP43 ALS mouse models. Hum Mol Genet.

[139] Verstreken P, Ly CV, Venken KJT, Koh T-W, Zhou Y, Bellen HJ (2005). Synaptic mitochondria are critical for mobilization of reserve pool vesicles at *Drosophila* neuromuscular junctions. Neuron.

[140] Lee CW, Peng HB (2008). The function of mitochondria in presynaptic development at the neuromuscular junction. Mol Biol Cell.

[141] Baldwin KR, Godena VK, Hewitt VL, Whitworth AJ (2016). Axonal transport defects are a common phenotype in *Drosophila* models of ALS. Hum Mol Genet.

[142] Orlacchio A, Babalini C, Borreca A, Patrono C, Massa R, Basaran S (2010). SPATACSIN mutations cause autosomal recessive juvenile amyotrophic lateral sclerosis. Brain.

[143] Brenner D, Yilmaz R, Müller K, Grehl T, Petri S, Meyer T (2018). Hot-spot KIF5A mutations cause familial ALS. Brain.

[144] Wu C-H, Fallini C, Ticozzi N, Keagle PJ, Sapp PC, Piotrowska K (2012). Mutations in the profilin 1 gene cause familial amyotrophic lateral sclerosis. Nature.

[145] Lalonde R, Strazielle C (2003). Neurobehavioral characteristics of mice with modified intermediate filament genes. Rev Neurosci.

[146] Pantelidou M, Zographos SE, Lederer CW, Kyriakides T, Pfaffl MW, Santama N (2007). Differential expression of molecular motors in the motor cortex of sporadic ALS. Neurobiol Dis.

[147] Castellanos-Montiel MJ, Chaineau M, Durcan TM (2020). The neglected genes of ALS: cytoskeletal dynamics impact synaptic degeneration in ALS. Front Cell Neurosci.

[148] Rotem N, Magen I, Ionescu A, Gershoni-Emek N, Altman T, Costa CJ (2017). ALS along the axons: expression of coding and noncoding RNA differs in axons of ALS models. Sci Rep.

[149] Briese M, Saal-Bauernschubert L, Lüningschrör P, Moradi M, Dombert B, Surrey V (2020). Loss of Tdp-43 disrupts the axonal transcriptome of motoneurons accompanied by impaired axonal translation and mitochondria function. Acta Neuropathol Commun.

[150] Narayanan RK, Mangelsdorf M, Panwar A, Butler TJ, Noakes PG, Wallace RH (2013). Identification of RNA bound to the TDP-43 ribonucleoprotein complex in the adult mouse brain. Amyotroph Lateral Scler Front Degener.

[151] Majumder P, Chu J-F, Chatterjee B, Swamy KBS, Shen C-KJ (2016). Co-regulation of mRNA translation by TDP-43 and Fragile X syndrome protein FMRP. Acta Neuropathol.

[152] Ishiguro A, Kimura N, Watanabe Y, Watanabe S, Ishihama A (2016). TDP-43 binds and transports G-quadruplex-containing mRNAs into neurites for local translation. Genes Cells.

[153] Zhang YQ, Bailey AM, Matthies HJG, Renden RB, Smith MA, Speese SD (2001). Drosophila Fragile X-related gene regulates the MAP1B homolog Futsch to control synaptic structure and function. Cell.

[154] Song C, Leahy SN, Rushton EM, Broadie K (2022). RNA-binding FMRP and staufen sequentially regulate the coracle scaffold to control synaptic glutamate receptor and bouton development. Development.

[155] Nagano S, Jinno J, Abdelhamid RF, Jin Y, Shibata M, Watanabe S (2020). TDP-43 transports ribosomal protein mRNA to regulate axonal local translation in neuronal axons. Acta Neuropathol.

[156] Nakata T, Terada S, Hirokawa N (1998). Visualization of the dynamics of synaptic vesicle and plasma membrane proteins in living axons. J Cell Biol.

[157] Zhai RG, Vardinon-Friedman H, Cases-Langhoff C, Becker B, Gundelfinger ED, Ziv NE (2001). Assembling the presynaptic active zone: a characterization of an active zone precursor vesicle. Neuron.

[158] Bronfman FC, Escudero CA, Weis J, Kruttgen A (2007). Endosomal transport of neurotrophins: roles in signaling and neurodegenerative diseases. Dev Neurobiol.

[159] Marqués G, Bao H, Haerry TE, Shimell MJ, Duchek P, Zhang B (2002). The *Drosophila* BMP type II receptor wishful thinking regulates neuromuscular synapse morphology and function. Neuron.

[160] McCabe BD, Marqués G, Haghighi AP, Fetter RD, Crotty ML, Haerry TE (2003). The BMP homolog gbb provides a retrograde signal that regulates synaptic growth at the *Drosophila* neuromuscular junction. Neuron.

[161] Nakamura M, Ito H, Wate R, Nakano S, Hirano A, Kusaka H (2008). Phosphorylated Smad2/3 immunoreactivity in sporadic and familial amyotrophic lateral sclerosis and its mouse model. Acta Neuropathol.

[162] Waller A (1850). Experiments on the section of the glossopharyngeal and hypoglossal nerves of the frog, and observations of the alterations produced thereby in the structure of their primitive fibres. Philos Trans R Soc Lond.

[163] Lubińska L (1977). Early course of wallerian degeneration in myelinated fibres of the rat phrenic nerve. Brain Res.

[164] Gerdts J, Summers DW, Sasaki Y, DiAntonio A, Milbrandt J (2013). Sarm1-mediated axon degeneration requires both SAM and TIR interactions. J Neurosci.

[165] Gerdts J, Brace EJ, Sasaki Y, DiAntonio A, Milbrandt J (2015). SARM1 activation triggers axon degeneration locally via NAD+ destruction. Science.

[166] Gilley J, Orsomando G, Nascimento-Ferreira I, Coleman MP (2015). Absence of SARM1 rescues development and survival of NMNAT2-deficient axons. Cell Rep.

[167] Gilley J, Ribchester RR, Coleman MP (2017). Sarm1 deletion, but not WldS, confers lifelong rescue in a mouse model of severe axonopathy. Cell Rep.

[168] Loreto A, Di Stefano M, Gering M, Conforti L (2015). Wallerian degeneration is executed by an NMN-SARM1-dependent late Ca^2+^ influx but only modestly influenced by mitochondria. Cell Rep.

[169] Osterloh JM, Yang J, Rooney TM, Fox AN, Adalbert R, Powell EH (2012). dSarm/Sarm1 is required for activation of an injury-induced axon death pathway. Science.

[170] Fogh I, Ratti A, Gellera C, Lin K, Tiloca C, Moskvina V (2014). A genome-wide association meta-analysis identifies a novel locus at 17q11.2 associated with sporadic amyotrophic lateral sclerosis. Hum Mol Genet.

[171] Gilley J, Jackson O, Pipis M, Estiar MA, Al-Chalabi A, Danzi MC (2021). Enrichment of SARM1 alleles encoding variants with constitutively hyperactive NADase in patients with ALS and other motor nerve disorders. Elife.

[172] Bloom AJ, Mao X, Strickland A, Sasaki Y, Milbrandt J, DiAntonio A (2022). Constitutively active SARM1 variants that induce neuropathy are enriched in ALS patients. Mol Neurodegener.

[173] Sajadi A (2004). Wlds-mediated protection of dopaminergic fibers in an animal model of Parkinson disease. Curr Biol.

[174] Tsai J, Grutzendler J, Duff K, Gan W-B (2004). Fibrillar amyloid deposition leads to local synaptic abnormalities and breakage of neuronal branches. Nat Neurosci.

[175] Galvin JE, Uryu K, Lee VMY, Trojanowski JQ (1999). Axon pathology in Parkinson’s disease and Lewy body dementia hippocampus contains α-, β-, and γ-synuclein. Proc Natl Acad Sci U S A.

[176] Stokin GB, Lillo C, Falzone TL, Brusch RG, Rockenstein E, Mount SL (2005). Axonopathy and transport deficits early in the pathogenesis of Alzheimer’s disease. Science.

[177] Li H, Li S-H, Yu Z-X, Shelbourne P, Li X-J (2001). Huntingtin aggregate-associated axonal degeneration is an early pathological event in Huntington’s disease mice. J Neurosci.

[178] Sasaki S, Iwata M (2007). Mitochondrial alterations in the spinal cord of patients with sporadic amyotrophic lateral sclerosis. J Neuropathol Exp Neurol.

[179] Wiedemann FR, Manfredi G, Mawrin C, Beal MF, Schon EA (2002). Mitochondrial DNA and respiratory chain function in spinal cords of ALS patients. J Neurochem.

[180] Borthwick GM, Johnson MA, Ince PG, Shaw PJ, Turnbull DM (1999). Mitochondrial enzyme activity in amyotrophic lateral sclerosis: implications for the role of mitochondria in neuronal cell death. Ann Neurol.

[181] Moisse K, Mepham J, Volkening K, Welch I, Hill T, Strong MJ (2009). Cytosolic TDP-43 expression following axotomy is associated with caspase 3 activation in NFL–/– mice: support for a role for TDP-43 in the physiological response to neuronal injury. Brain Res.

[182] Sato T, Takeuchi S, Saito A, Ding W, Bamba H, Matsuura H (2009). Axonal ligation induces transient redistribution of TDP-43 in brainstem motor neurons. Neuroscience.

[183] Moisse K, Volkening K, Leystra-Lantz C, Welch I, Hill T, Strong MJ (2009). Divergent patterns of cytosolic TDP-43 and neuronal progranulin expression following axotomy: implications for TDP-43 in the physiological response to neuronal injury. Brain Res.

[184] Swarup V, Audet JN, Phaneuf D, Kriz J, Julien JP (2012). Abnormal regenerative responses and impaired axonal outgrowth after nerve crush in TDP-43 transgenic mouse models of amyotrophic lateral sclerosis. J Neurosci.

[185] Vérièpe J, Fossouo L, Parker JA (2015). Neurodegeneration in C. elegans models of ALS requires TIR-1/Sarm1 immune pathway activation in neurons. Nat Commun.

[186] Velde C, Vande, Garcia ML, Yin X, Trapp BD, Cleveland DW (2004). The neuroprotective factor Wlds does not attenuate mutant SOD1-mediated motor neuron disease. NeuroMolecular Med.

[187] Peters OM, Lewis EA, Osterloh JM, Weiss A, Salameh JS, Metterville J (2018). Loss of Sarm1 does not suppress motor neuron degeneration in the SOD1G93A mouse model of amyotrophic lateral sclerosis. Hum Mol Genet.

[188] Marques RF, Engler JB, Küchler K, Jones RA, Lingner T, Salinas G (2020). Motor neuron translatome reveals deregulation of SYNGR4 and PLEKHB1 in mutant TDP-43 amyotrophic lateral sclerosis models. Hum Mol Genet.

[189] Klim JR, Williams LA, Limone F, Guerra San Juan I, Davis-Dusenbery BN, Mordes DA (2019). ALS-implicated protein TDP-43 sustains levels of STMN2, a mediator of motor neuron growth and repair. Nat Neurosci.

[190] Melamed Z, López-Erauskin J, Baughn MW, Zhang O, Drenner K, Sun Y (2019). Premature polyadenylation-mediated loss of stathmin-2 is a hallmark of TDP-43-dependent neurodegeneration. Nat Neurosci.

[191] Babetto E, Beirowski B, Russler EV, Milbrandt J, DiAntonio A (2013). The Phr1 ubiquitin ligase promotes injury-induced axon self-destruction. Cell Rep.

[192] Bloom AJ, Miller BR, Sanes JR, DiAntonio A (2007). The requirement for Phr1 in CNS axon tract formation reveals the corticostriatal boundary as a choice point for cortical axons. Genes Dev.

[193] Lewcock JW, Genoud N, Lettieri K, Pfaff SL (2007). The ubiquitin ligase Phr1 regulates axon outgrowth through modulation of microtubule dynamics. Neuron.

[194] Burgess RW, Peterson KA, Johnson MJ, Roix JJ, Welsh IC, O’Brien TP (2004). Evidence for a conserved function in synapse formation reveals Phr1 as a candidate gene for respiratory failure in newborn mice. Mol Cell Biol.

[195] Shin JE, Miller BR, Babetto E, Cho Y, Sasaki Y, Qayum S (2012). SCG10 is a JNK target in the axonal degeneration pathway. Proc Natl Acad Sci U S A.

[196] Shin JE, Geisler S, DiAntonio A (2014). Dynamic regulation of SCG10 in regenerating axons after injury. Exp Neurol.

[197] Graf ER, Heerssen HM, Wright CM, Davis GW, DiAntonio A (2011). Stathmin is required for stability of the *Drosophila* neuromuscular junction. J Neurosci.

[198] Duncan JE, Lytle NK, Zuniga A, Goldstein LSB (2013). The microtubule regulatory protein stathmin is required to maintain the integrity of axonal microtubules in *Drosophila*. PLoS ONE.

[199] Guerra San Juan I, Nash LA, Smith KS, Leyton-Jaimes MF, Qian M, Klim JR (2022). Loss of mouse Stmn2 function causes motor neuropathy. Neuron.

[200] Liedtke W, Leman EE, Fyffe REW, Raine CS, Schubart UK (2002). Stathmin-deficient mice develop an age-dependent axonopathy of the central and peripheral nervous systems. Am J Pathol.

[201] Johnson BS, Snead D, Lee JJ, McCaffery JM, Shorter J, Gitler AD (2009). TDP-43 is intrinsically aggregation-prone, and amyotrophic lateral sclerosis-linked mutations accelerate aggregation and increase toxicity. J Biol Chem.

[202] Ayala YM, Zago P, D’Ambrogio A, Xu YF, Petrucelli L, Buratti E (2008). Structural determinants of the cellular localization and shuttling of TDP-43. J Cell Sci.

[203] Coyne AN, Lorenzini I, Chou CC, Torvund M, Rogers RS, Starr A (2017). Post-transcriptional inhibition of Hsc70-4/HSPA8 expression leads to synaptic vesicle cycling defects in multiple models of ALS. Cell Rep.

[204] Kedersha N, Chen S, Gilks N, Li W, Miller IJ, Stahl J (2002). Evidence that ternary complex (eIF2-GTP-tRNAiMet)–deficient preinitiation complexes are core constituents of mammalian stress granules. Mol Biol Cell.

[205] Napoli L, Crugnola V, Lamperti C, Silani V, Di Mauro S, Bresolin N (2011). Ultrastructural mitochondrial abnormalities in patients with sporadic amyotrophic lateral sclerosis. Arch Neurol.

[206] Chung MJ, Suh Y-L (2002). Ultrastructural changes of mitochondria in the skeletal muscle of patients with amyotrophic lateral sclerosis. Ultrastruct Pathol.

[207] Afifi AK, Aleu FP, Goodgold J, MacKay B (1966). Ultrastructure of atrophic muscle in amyotrophic lateral sclerosis. Neurology.

[208] Crugnola V, Lamperti C, Lucchini V, Ronchi D, Peverelli L, Prelle A (2010). Mitochondrial respiratory chain dysfunction in muscle from patients with amyotrophic lateral sclerosis. Arch Neurol.

[209] Echaniz-Laguna A, Zoll J, Ponsot E, N’Guessan B, Tranchant C, Loeffler J-P (2006). Muscular mitochondrial function in amyotrophic lateral sclerosis is progressively altered as the disease develops: a temporal study in man. Exp Neurol.

[210] Vielhaber S, Winkler K, Kirches E, Kunz D, Büchner M, Feistner H (1999). Visualization of defective mitochondrial function in skeletal muscle fibers of patients with sporadic amyotrophic lateral sclerosis. J Neurol Sci.

[211] Wiedemann FR, Winkler K, Kuznetsov AV, Bartels C, Vielhaber S, Feistner H (1998). Impairment of mitochondrial function in skeletal muscle of patients with amyotrophic lateral sclerosis. J Neurol Sci.

[212] Lunetta C, Serafini M, Prelle A, Magni P, Dozio E, Ruscica M (2012). Impaired expression of insulin-like growth factor‐1 system in skeletal muscle of amyotrophic lateral sclerosis patients. Muscle Nerve.

[213] Yamamoto M, Sobue G, Yamamoto K, Terao S, Mitsuma T (1996). Expression of glial cell line-derived growth factor mRNA in the spinal cord and muscle in amyotrophic lateral sclerosis. Neurosci Lett.

[214] Bruneteau G, Bauché S, Gonzalez de Aguilar JL, Brochier G, Mandjee N, Tanguy M-L (2015). Endplate denervation correlates with Nogo-A muscle expression in amyotrophic lateral sclerosis patients. Ann Clin Transl Neurol.

[215] Koistinen H, Prinjha R, Soden P, Harper A, Banner SJ, Pradat P-F (2006). Elevated levels of amyloid precursor protein in muscle of patients with amyotrophic lateral sclerosis and a mouse model of the disease. Muscle Nerve.

[216] Jokic N, Gonzalez de Aguilar J-L, Pradat P-F, Dupuis L, Echaniz-Laguna A, Muller A (2005). Nogo expression in muscle correlates with amyotrophic lateral sclerosis severity. Ann Neurol.

[217] D’Agostino C, Nogalska A, Engel WK, Askanas V (2011). In sporadic inclusion body myositis muscle fibres TDP-43-positive inclusions are less frequent and robust than p62 inclusions, and are not associated with paired helical filaments. Neuropathol Appl Neurobiol.

[218] Weihl CC, Temiz P, Miller SE, Watts G, Smith C, Forman M (2008). TDP-43 accumulation in inclusion body myopathy muscle suggests a common pathogenic mechanism with frontotemporal dementia. J Neurol Neurosurg Psychiatry.

[219] Campanari M-L, Bourefis A-R, Kabashi E (2019). Diagnostic challenge and neuromuscular junction contribution to ALS pathogenesis. Front Neurol.

[220] Mori F, Tada M, Kon T, Miki Y, Tanji K, Kurotaki H (2019). Phosphorylated TDP-43 aggregates in skeletal and cardiac muscle are a marker of myogenic degeneration in amyotrophic lateral sclerosis and various conditions. Acta Neuropathol Commun.

[221] Cykowski MD, Powell SZ, Appel JW, Arumanayagam AS, Rivera AL, Appel SH (2018). Phosphorylated TDP-43 (pTDP-43) aggregates in the axial skeletal muscle of patients with sporadic and familial amyotrophic lateral sclerosis. Acta Neuropathol Commun.

[222] Hernandez Lain A, Millecamps S, Dubourg O, Salachas F, Bruneteau G, Lacomblez L (2011). Abnormal TDP-43 and FUS proteins in muscles of sporadic IBM: similarities in a TARDBP-linked ALS patient. J Neurol Neurosurg Psychiatry.

[223] Vogler TO, Wheeler JR, Nguyen ED, Hughes MP, Britson KA, Lester E (2018). TDP-43 and RNA form amyloid-like myo-granules in regenerating muscle. Nature.

[224] Militello G, Hosen MR, Ponomareva Y, Gellert P, Weirick T, John D (2018). A novel long non-coding RNA myolinc regulates myogenesis through TDP-43 and Filip1. J Mol Cell Biol.

[225] Tawara N, Yamashita S, Kawakami K, Kurashige T, Zhang Z, Tasaki M (2018). Muscle-dominant wild-type TDP-43 expression induces myopathological changes featuring tubular aggregates and TDP-43-positive inclusions. Exp Neurol.

[226] Schmid B, Hruscha A, Hogl S, Banzhaf-Strathmann J, Strecker K, van der Zee J (2013). Loss of ALS-associated TDP-43 in zebrafish causes muscle degeneration, vascular dysfunction, and reduced motor neuron axon outgrowth. Proc Natl Acad Sci U S A.

[227] Diaper DC, Adachi Y, Lazarou L, Greenstein M, Simoes FA, Di Domenico A (2013). Drosophila TDP-43 dysfunction in glia and muscle cells cause cytological and behavioural phenotypes that characterize ALS and FTLD. Hum Mol Genet.

[228] Scaramozza A, Marchese V, Papa V, Salaroli R, Sorarù G, Angelini C (2014). Skeletal muscle satellite cells in amyotrophic lateral sclerosis. Ultrastruct Pathol.

[229] Pradat P-F, Barani A, Wanschitz J, Dubourg O, Lombès A, Bigot A (2011). Abnormalities of satellite cells function in amyotrophic lateral sclerosis. Amyotroph Lateral Scler.

[230] Kohsaka H, Takasu E, Nose A (2007). In vivo induction of postsynaptic molecular assembly by the cell adhesion molecule Fasciclin2. J Cell Biol.

[231] Zito K, Fetter RD, Goodman CS, Isacoff EY (1997). Synaptic clustering of Fasciclin II and Shaker: essential targeting sequences and role of Dlg. Neuron.

[232] Thomas U, Kim E, Kuhlendahl S, Koh YH, Gundelfinger ED, Sheng M (1997). Synaptic clustering of the cell adhesion molecule fasciclin II by discs-large and its role in the regulation of presynaptic structure. Neuron.

[233] Lynch EM, Robertson S, FitzGibbons C, Reilly M, Switalski C, Eckardt A (2021). Transcriptome analysis using patient iPSC-derived skeletal myocytes: Bet1L as a new molecule possibly linked to neuromuscular junction degeneration in ALS. Exp Neurol.

[234] King IN, Yartseva V, Salas D, Kumar A, Heidersbach A, Ando DM (2014). The RNA-binding protein TDP-43 selectively disrupts microRNA-1/206 incorporation into the RNA-induced silencing complex. J Biol Chem.

[235] Chen J-F, Mandel EM, Thomson JM, Wu Q, Callis TE, Hammond SM (2006). The role of microRNA-1 and microRNA-133 in skeletal muscle proliferation and differentiation. Nat Genet.

[236] Ivey KN, Muth A, Arnold J, King FW, Yeh R-F, Fish JE (2008). MicroRNA regulation of cell lineages in mouse and human embryonic stem cells. Cell Stem Cell.

[237] Simon DJ, Madison JM, Conery AL, Thompson-Peer KL, Soskis M, Ruvkun GB (2008). The microRNA miR-1 regulates a MEF-2-dependent retrograde signal at neuromuscular junctions. Cell.

[238] Williams AH, Valdez G, Moresi V, Qi X, McAnally J, Elliott JL (2009). MicroRNA-206 delays ALS progression and promotes regeneration of neuromuscular synapses in mice. Science.

[239] Nagel G, Peter RS, Rosenbohm A, Koenig W, Dupuis L, Rothenbacher D (2020). Association of insulin-like growth factor 1 concentrations with risk for and prognosis of amyotrophic lateral sclerosis: results from the ALS registry Swabia. Sci Rep.

[240] Bruneteau G, Simonet T, Bauché S, Mandjee N, Malfatti E, Girard E (2013). Muscle histone deacetylase 4 upregulation in amyotrophic lateral sclerosis: potential role in reinnervation ability and disease progression. Brain.

[241] Di Pietro L, Baranzini M, Berardinelli MG, Lattanzi W, Monforte M, Tasca G (2017). Potential therapeutic targets for ALS: MIR206, MIR208b and MIR499 are modulated during disease progression in the skeletal muscle of patients. Sci Rep.

[242] Wächter N, Storch A, Hermann A, Human (2015). TDP-43 and FUS selectively affect motor neuron maturation and survival in a murine cell model of ALS by non-cell-autonomous mechanisms. Amyotroph Lateral Scler Front Degener.

[243] Maimon R, Ionescu A, Bonnie A, Sweetat S, Wald-Altman S, Inbar S (2018). miR126-5p downregulation facilitates axon degeneration and NMJ disruption via a non–cell-autonomous mechanism in ALS. J Neurosci.

[244] Ishii T, Kawakami E, Endo K, Misawa H, Watabe K (2017). Formation and spreading of TDP-43 aggregates in cultured neuronal and glial cells demonstrated by time-lapse imaging. PLoS ONE.

[245] Haidet-Phillips AM, Hester ME, Miranda CJ, Meyer K, Braun L, Frakes A (2011). Astrocytes from familial and sporadic ALS patients are toxic to motor neurons. Nat Biotechnol.

[246] Rojas F, Cortes N, Abarzua S, Dyrda A, van Zundert B (2014). Astrocytes expressing mutant SOD1 and TDP43 trigger motoneuron death that is mediated via sodium channels and nitroxidative stress. Front Cell Neurosci.

[247] Huang C, Huang B, Bi F, Yan LH, Tong J, Huang J (2014). Profiling the genes affected by pathogenic TDP-43 in astrocytes. J Neurochem.

[248] LaRocca TJ, Mariani A, Watkins LR, Link CD (2019). TDP-43 knockdown causes innate immune activation via protein kinase R in astrocytes. Neurobiol Dis.

[249] Deora V, Lee JD, Albornoz EA, McAlary L, Jagaraj CJ, Robertson AAB (2020). The microglial NLRP3 inflammasome is activated by amyotrophic lateral sclerosis proteins. Glia.

[250] Ke YD, van Hummel A, Stevens CH, Gladbach A, Ippati S, Bi M (2015). Short-term suppression of A315T mutant human TDP-43 expression improves functional deficits in a novel inducible transgenic mouse model of FTLD-TDP and ALS. Acta Neuropathol.

[251] Jara JH, Gautam M, Kocak N, Xie EF, Mao Q, Bigio EH (2019). MCP1-CCR2 and neuroinflammation in the ALS motor cortex with TDP-43 pathology. J Neuroinflamm.

[252] Lee JD, Levin SC, Willis EF, Li R, Woodruff TM, Noakes PG (2018). Complement components are upregulated and correlate with disease progression in the TDP-43 Q331K mouse model of amyotrophic lateral sclerosis. J Neuroinflamm.

[253] Xu Y-F, Zhang Y-J, Lin W-L, Cao X, Stetler C, Dickson DW (2011). Expression of mutant TDP-43 induces neuronal dysfunction in transgenic mice. Mol Neurodegener.

[254] Lee S, Kim S, Kang HY, Lim HR, Kwon Y, Jo M (2020). The overexpression of TDP-43 in astrocytes causes neurodegeneration via a PTP1B-mediated inflammatory response. J Neuroinflamm.

[255] Peng AYT, Agrawal I, Ho WY, Yen YC, Pinter AJ, Liu J (2020). Loss of TDP-43 in astrocytes leads to motor deficits by triggering A1-like reactive phenotype and triglial dysfunction. Proc Natl Acad Sci U S A.

[256] Wang J, Ho WY, Lim K, Feng J, Tucker-Kellogg G, Nave K-A (2018). Cell-autonomous requirement of TDP-43, an ALS/FTD signature protein, for oligodendrocyte survival and myelination. Proc Natl Acad Sci U S A.

[257] Ho WY, Chang J-C, Lim K, Cazenave-Gassiot A, Nguyen AT, Foo JC (2021). TDP-43 mediates SREBF2-regulated gene expression required for oligodendrocyte myelination. J Cell Biol.

[258] Heo D, Ling JP, Molina-Castro GC, Langseth AJ, Waisman A, Nave K-A (2022). Stage-specific control of oligodendrocyte survival and morphogenesis by TDP-43. Elife.

[259] Rothstein JD, Van Kammen M, Levey AI, Martin LJ, Kuncl RW (1995). Selective loss of glial glutamate transporter GLT-1 in amyotrophic lateral sclerosis. Ann Neurol.

[260] Tong J, Huang C, Bi F, Wu Q, Huang B, Liu X (2013). Expression of ALS-linked TDP-43 mutant in astrocytes causes non-cell-autonomous motor neuron death in rats. EMBO J.

[261] Colombo MN, Francolini M (2019). Glutamate at the vertebrate neuromuscular junction: from modulation to neurotransmission. Cells.

[262] Liou HC, Yang RS, Fu WM (1996). Potentiation of spontaneous acetylcholine release from motor nerve terminals by glutamate in Xenopus tadpoles. Neuroscience.

[263] Riva N, Gentile F, Cerri F, Gallia F, Podini P, Dina G (2022). Phosphorylated TDP-43 aggregates in peripheral motor nerves of patients with amyotrophic lateral sclerosis. Brain.

[264] Perrie WT, Lee GT, Curtis EM, Sparke J, Buller JR, Rossi ML (1993). Changes in the myelinated axons of femoral nerve in amyotrophic lateral sclerosis. J Neural Transm Suppl.

[265] Chang KJ, Agrawal I, Vainshtein A, Ho WY, Xin W, Tucker-Kellogg G (2021). TDP-43 maximizes nerve conduction velocity by repressing a cryptic exon for paranodal junction assembly in Schwann cells. Elife.

[266] Nakamura-Shindo K, Sakai K, Shimizu A, Ishida C, Yamada M (2020). Accumulation of phosphorylated TDP-43 in the cytoplasm of Schwann cells in a case of sporadic amyotrophic lateral sclerosis. Neuropathology.

[267] de Carvalho M (2020). Electrodiagnosis of amyotrophic lateral sclerosis: a review of existing guidelines. J Clin Neurophysiol.

[268] Sanes JR, Lichtman JW (1999). Development of the vertebrate neuromuscular junction. Annu Rev Neurosci.

[269] Feng Z, Ko C-P (2008). The role of glial cells in the formation and maintenance of the neuromuscular junction. Ann N Y Acad Sci.

[270] Reddy LV, Koirala S, Sugiura Y, Herrera AA, Ko C-P (2003). Glial cells maintain synaptic structure and function and promote development of the neuromuscular junction in vivo. Neuron.

[271] Bélair E-L, Vallée J, Robitaille R (2010). In vivo long-term synaptic plasticity of glial cells. J Physiol.

[272] Son Y-J, Thompson WJ (1995). Schwann cell processes guide regeneration of peripheral axons. Neuron.

[273] Zuo Y (2004). Fluorescent proteins expressed in mouse transgenic lines mark subsets of glia, neurons, macrophages, and dendritic cells for vital examination. J Neurosci.

[274] Son Y-J, Thompson WJ (1995). Nerve sprouting in muscle is induced and guided by processes extended by schwann cells. Neuron.

[275] Arbour D, Tremblay E, Martineau É, Julien J-P, Robitaille R (2015). Early and persistent abnormal decoding by glial cells at the neuromuscular junction in an ALS model. J Neurosci.

[276] Carrasco DI, Seburn KL, Pinter MJ (2016). Altered terminal Schwann cell morphology precedes denervation in SOD1 mice. Exp Neurol.

[277] Martineau É, Arbour D, Vallée J, Robitaille R (2020). Properties of glial cell at the neuromuscular junction are incompatible with synaptic repair in the SOD1G37R ALS mouse model. J Neurosci.

[278] Rochon D, Rousse I, Robitaille R (2001). Synapse–glia interactions at the mammalian neuromuscular junction. J Neurosci.

[279] Masrori P, Van Damme P (2020). Amyotrophic lateral sclerosis: a clinical review. Eur J Neurol.

